# Signaling pathways and intervention therapies in sepsis

**DOI:** 10.1038/s41392-021-00816-9

**Published:** 2021-11-25

**Authors:** Yun-yu Zhang, Bo-tao Ning

**Affiliations:** Department of Pediatric Intensive Care Unit, Shanghai Children’s Medical Center, School of Medicine, Shanghai Jiao Tong University, 200127 Shanghai, China

**Keywords:** Infection, Infectious diseases, Inflammation

## Abstract

Sepsis is defined as life-threatening organ dysfunction caused by dysregulated host systemic inflammatory and immune response to infection. Over decades, advanced understanding of host–microorganism interaction has gradually unmasked the genuine nature of sepsis, guiding toward new definition and novel therapeutic approaches. Diverse clinical manifestations and outcomes among infectious patients have suggested the heterogeneity of immunopathology, while systemic inflammatory responses and deteriorating organ function observed in critically ill patients imply the extensively hyperactivated cascades by the host defense system. From focusing on microorganism pathogenicity, research interests have turned toward the molecular basis of host responses. Though progress has been made regarding recognition and management of clinical sepsis, incidence and mortality rate remain high. Furthermore, clinical trials of therapeutics have failed to obtain promising results. As far as we know, there was no systematic review addressing sepsis-related molecular signaling pathways and intervention therapy in literature. Increasing studies have succeeded to confirm novel functions of involved signaling pathways and comment on efficacy of intervention therapies amid sepsis. However, few of these studies attempt to elucidate the underlining mechanism in progression of sepsis, while other failed to integrate preliminary findings and describe in a broader view. This review focuses on the important signaling pathways, potential molecular mechanism, and pathway-associated therapy in sepsis. Host-derived molecules interacting with activated cells possess pivotal role for sepsis pathogenesis by dynamic regulation of signaling pathways. Cross-talk and functions of these molecules are also discussed in detail. Lastly, potential novel therapeutic strategies precisely targeting on signaling pathways and molecules are mentioned.

## Introduction

In the past 50 years, sepsis was being defined as the development of host systemic inflammatory response syndrome (SIRS) against microbial infection. The term “SIRS” referred as a clinical syndrome featured with systemic inflammation and extensive tissue injuries. Diagnosis could be confirmed after fulfilling at least two of the following clinical criteria: tachypnea (rapid breathing), tachycardia (rapid heartbeat), pyrexia (fever) or hypothermia, and leukocytosis or leukopenia. With advanced knowledge and initiatives to speed up global awareness of sepsis and to build collaboration between experts groups, Society of Critical Care Medicine (SCCM) and European Society of Intensive Care Medicine (ESICM) jointly initiated the Surviving Sepsis Campaign (SSC) in 2001.^1^ The global sepsis consensus established in 2004 has speeded up formulation of a standard definition for sepsis (also known as sepsis-2).^2^ This international task force reviewed and updated the sepsis guideline every 4 years.

Over years, advanced understanding of sepsis pathophysiology has revealed its nature, implicating that sepsis should not be defined simply as syndrome of inflammation. Evidently, the SIRS concept was too broadly applied to critically ill patients and inadequate to meet the clinical heterogeneity and dynamic changes in actual scenario. In the year of 2016, a revised international guideline with modified definition and diagnostic criteria of sepsis and septic shock (sepsis-3) was established, supported with evidenced-based recommendations for improved recognition and appropriate management of sepsis.^3^ Conceptually, sepsis is now defined as life-threatening organ dysfunction caused by a dysregulated host response against infection, while septic shock is defined as a subset of sepsis patients accompanied with circulatory and cellular/metabolic dysfunction. To better implement this definition, quick assessment tool such as SOFA (sequential organ failure assessment) was introduced to grade sepsis severity and predict in-hospital mortality based on well-defined multi-physiological criteria.

Soon after the pre-existed conceptual definition of adult sepsis, definitions for pediatric sepsis was introduced in 2005.^4^ In consideration of the distinct physiological features and organ function within each developmental stage, age-adjusted range for main clinical variables were provided in this guideline. In 2017, the American College of Critical Care Medicine proposed a standardized guideline for hemodynamic management of neonatal and pediatric septic shock.^5^ By reviewing efficacy of various clinical management procedures mentioned in pre-existed studies, guideline recommended implementation of a goal-directed strategy in resource-rich settings and have provided step-wise management tools for practical use. To counteract with the fundamental pathophysiological changes in sepsis, this guideline shed lights on the importance of hemodynamic stabilization and listed supporting procedures for hemodynamics management.

Even though global consensus guideline for sepsis was revised timely, recent version lack discussion regarding management of pediatric sepsis. In response, SCCM and ESICM published an updated statement guideline for management of septic shock and septic organ failure in children.^6^ Evidence-based recommendations responding to concerns in screening, diagnosis, and treatment of pediatric sepsis were provided to guide “best clinical practice.” Though definition for pediatric sepsis is still pending for renew, concept of sepsis-3 was implemented throughout formulation of these recommendations.

Sepsis remains a burdensome public health problem globally. In a tentative exploration of hospital-treated sepsis data obtained from high-income countries, a global estimate of 31.5 million sepsis cases were reported, with potentially 5.3 million deaths annually.^7^ Indeed, sepsis were considered to be a huge burden of out-hospital deaths in low-income countries, due to the high prevalence of infectious disease such as malaria, HIV, and dengue fever.^8,9^ In view of this, a recent study has devoted to provide accurate global sepsis estimates by analyzing both in and out-hospital sepsis-related death among 195 countries. In the year of 2017, an estimated population of 48.9 million were affected by sepsis worldwide. Though mortality rate has decreased by 52.8% from 1990 to 2017, the reported 11.0 million sepsis-related deaths annually still account for 19.7% of the global deaths.^10^ Moreover, sepsis shows significant higher burden in regions with poorer access to medical care such as sub-Saharan Africa and Southeast Asia, thus explaining discrepancies among epidemiological studies due to study data limited to high- or low-income countries.

In the same report, more than half of sepsis estimates (25.2 million) were aged <20 years, which is substantially higher than previous estimates based on data from high-income countries.^11^ In the global burden of disease study, infection-related deaths have contributed as the second leading cause for childhood deaths.^12^ As only types of infection rather than the mutual pathophysiological event “sepsis” were included as causes of death in this study, incidence of sepsis-related deaths might be underestimated. Though incidence of early-onset neonatal sepsis has significantly declined in the past decades following implementation of administrating prophylaxis antibiotics and parental screening in high-income countries,^13,14^ sepsis remains a leading challenge in pediatric population in less-developed countries.

In a latest global survey of pediatric sepsis, severe sepsis remains a leading cause of death for critically ill patients in pediatric intensive care unit (PICU) within United States, accounting for about one-third of PICU deaths with a mortality rate of 25%.^15^ In previously healthy pediatric sepsis patients, 75% deaths events occurred within 3 days from sepsis recognition. As one of the most prevalent critical condition in PICU, refractory septic shock was indicated with a mortality rate of 34% and have accounted to one-thirds of early deaths.^16^ This mortality rate exceeds the prior estimates that relied on retrospective studies. Escalating incidence of sepsis might be attributed to the increased population with chronic critical illness due to improved healthcare services. However, prolonged hospitalization and frequent invasive procedures have increased risk for nosocomial infection, while intensive medications and immunosuppressive state might contribute to multidrug-resistant organism and opportunistic infection, together with the long-term outcomes such as organ failure, immunosuppression, and disabilities, posing an unprecedented great challenge for pediatric management. To cast light on sepsis pathogenesis and novel intervention therapeutic results, we systemically reviewed relevant studies and focused on the molecular signaling pathways and intervention therapies in sepsis.

## Signaling pathways in sepsis

Involvement of intricate signaling pathways and dysregulated host response makes sepsis a life-threatening heterogeneous syndrome different from mild infection. The initiating event in sepsis was host recognition of microbial-derived pathogen-associated molecular patterns (PAMPs) or endogenous damage-associated molecular patterns (DAMPs), guaranteed by a series of pattern recognition receptors (PRRs) located at cell membrane or intracellular space. Recognition result in the activation of intracellular signaling pathways. PAMPs and DAMPs range from microbial products, host glycoproteins, lipoproteins, and nucleic acids. In reciprocal, DAMPs and PAMPs bind and interact with Toll-like receptors (TLRs), C-type lectin domain family 7 member A (dectin 1) and C-type lectin domain family 6 member A (dectin 2). Once activated, the subsequent signaling pathways converge toward interferon regulatory factor (IRF) and nuclear factor-κB (NF-κB). IRF is responsible for type I interferon (IFN) production. NF-κB and activator protein 1 (AP-1) signaling are responsible for the early activation of inflammatory genes and those encoding endothelial cell-surface molecules. Immune cells could respond and interact with complexed intracellular signaling system to elicit innate immune responses for elimination of invading pathogens and cell homeostasis, while during sepsis, some of these involved host signaling pathways were abruptly upregulated with robust release of cytokines and alarmins (DAMPs) that serve as potent mediators for excessive systemic inflammation and immune suppression. Modulation and reprogramming of other essential signaling pathways under cellular stress took part to sustain effective innate immune response might also set off detrimental effects.

### Cytosolic sensing: STING-IRF3-NF-κB pathway

**Fig. 1:**
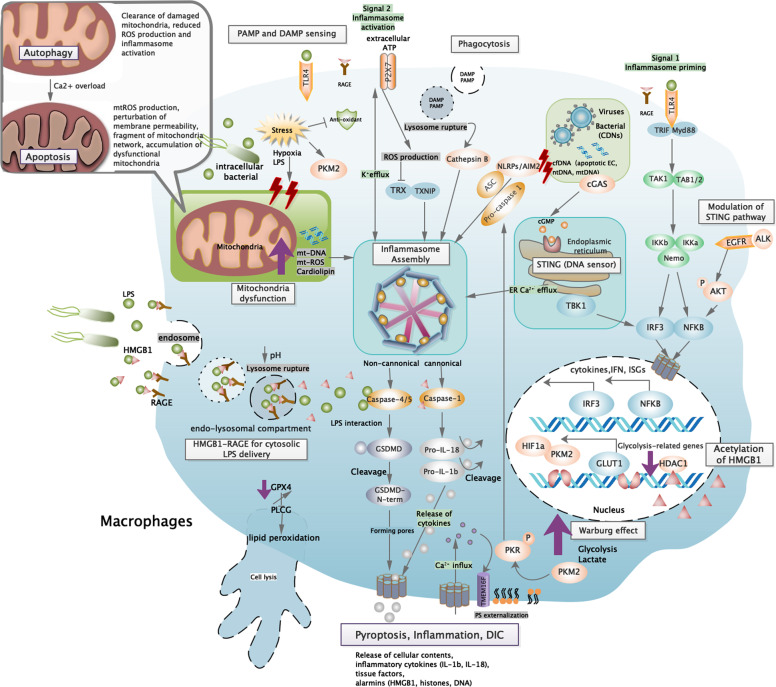
Cross-talks of signaling pathway in innate immune cells. At initiation of sepsis, innate immune cells are generally activated at recognition of DAMPs and PAMPs. Membrane-bound and intracellular receptors sense danger signals, which converge with multiple pathways related to activation and regulation of innate immune responses. In general, these converge toward IRF3 and NF-κB signaling pathway that are required to initiate early phase inflammatory responses. Besides, TLR4 agonist (i.e., LPS or HMGB1) provide essential priming signal for the first step of inflammasome activation—upregulation of pro-inflammatory genes. Another substantial group of pathogenic products and endogenous alarmins were required to provide signal 2 for AIM2/NLRP3 inflammasome assembly, which subsequently cleave caspase, GSDMD, and pro-IL-1β/18 to drive canonical inflammasome activation and pyroptosis. Acting as late-phase alarmin, HMGB1 interact with RAGE for delivery of cytosolic LPS, which further trigger pyroptosis via caspase-11-dependent pathway (equivalent to caspase-4 and caspase-5 in human, non-canonical pathway). Intracellularly, cytosolic DNA derived from apoptotic cells or intracellular pathogen could be sensed by AIM2 and cGAS-STING to induce inflammasome assembly and IRF3 phosphorylation, leading to type I interferon responses and inflammasome activation. During sepsis, persisted stress stimuli result in mitochondria dysfunction, ROS production, and metabolism reprogramming that further enhance redox state modification and alarmin production (HMGB1). These cellular changes activate multiple signaling pathways that converge with NLRP3 inflammasome activation. Inflammasome plays a pivotal role in sepsis pathogenesis due to its cross-talk with stress signaling, immune cell activation, and cell homeostasis. Interaction between inflammatory and coagulation cascades serve as underlined mechanism for DIC pathogenesis. As a cytosolic LPS receptor, caspase-11 participated in initiation of coagulation by enhancing tissue factor (TF) activity, performed by GSDMD pore-mediated Ca^2+^ efflux that trigger PS externalization, while macrophage pyroptosis facilitate release of cellular contents including procoagulant products (TF) and alarmins to sustain systemic coagulation. ROS reactive oxygen species, DAMP damage-associated molecular pattern, PAMP pathogen-associated molecular pattern, cfDNA cell-free DNA, EC endothelial cells, ER endoplasmic reticulum, ASC apoptosis-associated speck-like protein, TRX thioredoxin, TXNIP thioredoxin-interacting protein, HMGB1 high mobility group box protein 1, RAGE receptor for advanced glycation end products, GPX4 glutathione peroxidase 4, PLCG phospholipase C gamma 1, PKM2 pyruvate kinase M2, PKR RNA-activated protein kinase, HDAC1 histone deacetylase 1, GLUT1 glucose transporter 1, HIF1a hypoxia-inducible factor-1, PS phosphatidylserine, GSDMD gasdermin D

During sepsis, several types of host plasma cell-free DNA (cfDNA) were significantly elevated. Hepatocyte-derived cfDNA and cardiomyocyte cfDNA were passively released due to tissue damages.^17,18^ Compared to nuclear DNA (nDNA) derived from neutrophils, elevated levels of mitochondrial DNA (mtDNA) were shown to be associated with septic shock and mortality.^19^ cfDNA were potent to mediate inflammation and immune responses via interacting with STING (Fig. 1). In mice, the impaired ability to degrade cfDNA due to loss of deoxyribonuclease (DNase) could result in massive inflammation.^20^ Analogously, administration of DNase could improve survival via cleavage of cfDNA, therefore inhibiting the pro-inflammatory effect mediated by nDNA from neutrophil activation or NETosis.^21^

It was previously manifested that inappropriate digested apoptotic DNA accumulated in lysosomal compartments were potent to stimulate production of type I IFNs in a STING-dependent manner. This phenomenon could be observed in engulfed apoptotic or necrotic cells and serve as vehicles for delivery of cfDNA to cytosol.^22^ Other studies propose that extracellular vesicles such as apoptosis-derived membrane vesicles and exosomes participated in delivery of cfDNA to immune cells, which enhances type I IFN through cGAS-STING pathway.^23^

With established role as an adapter protein during IFN induction in response to cytosolic DNA sensing, the endoplasmic reticulum (ER)-bounded STING also serve as innate immune sensor for bacterial cyclic dinucleotides (CDNs), a group of conserved signaling molecules specifically released by bacteria. CDNs (c-di-GMP or c-di-AMP) gain access into immune cells and directly bind with STING.^24^ As a consequence, STING forms activated homodimer and was facilitated to form oligomeric state that lead to phosphorylation of downstream TANK-binding kinase 1 (TBK1) dimers, which consequently activate IRF3 and NF-κB signaling pathway.^25,26^ In lipopolysaccharide (LPS)-induced cardiac dysfunction model, LPS stimulation mediated by TLR4 is observed to induce STING perinuclear translocation, downstream IRF3 phosphorylation, and NLRP3 expression, while knockdown of STING in mice could alleviate sepsis-induced cardiac dysfunction with better survival rate via suppressing myocardial and serum inflammatory cytokines, apoptosis, and pyroptosis.^27^ STING-mediated cytokines also showed diverse immunoregulatory role via activating Janus-activated kinase (JAK)-signal transducer and activator of transcription factor (STAT) pathway.

### Complement system

Activation of complement system marked the prelude of host recognition of hazardous signals and cascades of defensive signaling pathways related to inflammation, coagulation, and bacterial cell lysis. These distinct properties of complements regulate early innate immune responses and are essential for protecting host from uncontrolled dissemination of invasive pathogens. The complement cascade was known to be activated via three separate pathways: classical pathway in response to antigen–antibody complex, alternative pathway by factor B/D, and lectin pathway via recognition of pathogen-derived mannose (by host mannose-binding lectin (MBL)). C3 act as the central component that all three pathways converge. Activating stimuli were classified into two main types: (1) pathogen-derived products (PAMP) such as LPS, mannose, and antigen–antibody complex; (2) endogenous cellular debris (DAMP) resulted from disturbances of cell homeostasis. These pattern signals were detected during initiating phase and assembly to propagate cleavage of complements. Cascade of complement cleavage and assembly result in formation of catalytic C3 convertase (C4b2b). C3 was then cleaved to obtain excess C3b that amplifies complement response. Accumulation of C3 cleavage products covalently bound to pathogen surface and facilitate phagocytosis by neutrophils, an antimicrobial process specifically known as opsonization. Adequate surface density of C3b subsequently lead to functional shift from C3 to C5 convertase. C5 convertase marked the formation of terminal C5b-9 complement complex—also known as membrane attack complex (MAC). Incorporation of MAC into pathogen surfaces create pores in the bacterial cell wall that induce cell lysis and guarantee clearance of specific type of intracellular bacterial (i.e., Neisseria species), while anaphylatoxins C3a and C5a are potent in coordinating with various inflammatory responses via directly binding to reciprocal receptors so to allow recruitment and activation of innate immune cells (neutrophils, monocytes, and macrophages).

Complement-mediated neutrophil activation is not only responsible for various prominent effector events in sepsis but also plays an ambivalent role in innate defense. Complement activation products C3a, C5a, and C5b interact with cell membrane receptors to induce antimicrobial response and pro-inflammatory effect via cross-talk with multiple signaling pathways. Extracellular signal-regulated kinase (ERK1/2) and p38-mitogen-activated protein kinase (MAPK) might be involved in the generation of interleukin (IL)-6 in neutrophils,^28^ while phosphatidylinositide 3-kinase (PI3K) control C5a-mediated response by regulating oxidative burst of neutrophils and macrophages as well as phagocytosis activity in neutrophils.^29^ C5a also facilitate phosphorylation and translocation of dormant intracellular enzyme (p47phox) so to induce activation of NADPH oxidase complex and generate oxidative burst for pathogen killing.^30^ However, sustained neutrophil activation might exhibit excessive response that are pathogenic, resulting in increased risks of disseminated infection and tissue damages.

As complement activation proceed, sustained generation of C5a might lead to paralysis of innate immune, resulting in dampened inflammatory and bacterial-killing effect.^30^ Such suppressed responses were mainly observed in neutrophils. Apparently, reduced tumor necrosis factor (TNF) secretion by neutrophils could be resulted from inhibition of NF-κB transcription due to increased levels of C5a-mediated IκBα.^31^ Besides, high levels of C5a might result in less targeted migration property of neutrophils.^32^ Disruption of C3a–C3aR axis by C5a promote egress of premature granulocytes and hematopoietic stem cells from bone marrow, presented with less targeted but more progressive inflammatory responses.^33^ C5a signal could simultaneously downregulate CXCR4 on granulocytes and facilitate release of protease that causes matrix protein degradation and inhibition of homing effect of stroma cell-derived factor 1, resulting in altered neutrophil phenotype.^34^

The direct activation of complement components by certain coagulation protease were known as “extrinsic protease pathway.”^35^ Thrombin potentially generate active C5a in the absence of C3 and might substitute as an independent novel complement pathway,^36^ while plasmin, kallikrein, and Hageman factor fragment were potent C3 and C5 convertase in in vitro studies and these biological fragments were related with increased chemotactic activity and cell proliferation.^37–39^ Aside from protease, pentraxins (CRP, SAP, PTX3) released in response to infection were potent in initiating classical pathway via interaction with C1q. Such mechanism was implicated to remove cellular debris during infection or tissue injuries.^40^

### Stress signaling mediated via reactive oxygen species (ROS)/NLRP3 inflammasome activation

Stress signaling is a highly conserved mechanism essential for exhibiting host defense response. Capable in sensing harmful signals (whole pathogen, PAMPs, environmental irritants) and damage-associated alarmins (extracellular ATP), stress signaling engage with pathways associated with appropriate cellular repairing and usually followed by a general scheme—stress stimuli detected by sensor proteins, oligomerization of sensor that allowed subsequent recruitment of effector protein, and finally activation of effector proteins for repairing responses.^41^ NLRP3 inflammasome assembly is a well-known sensing model that function to modulate such protective innate immune response during sepsis.^42^ In reviewing underlying signaling pathways and mechanisms in massive candidates of NLRP3 activators, ROS is noted as a group of crucial intermediators for engaging stress sensors and effectors due to its ability in integrating multiple signaling pathways associated with NLRP3 inflammasomes and innate immune responses.^43^

Injury-released extracellular ATP trigger generation of short-lived ROS via a pore-forming channel mechanism coupled with P2X7 receptor activation (Fig. 1), while large particulates (silica, alum) produce ROS depending on lysosome rupture process amid phagocytosis (Fig. 1).^44,45^ When confronting cellular stress, TLR-mediated mitochondria ROS and NADPH oxidase generation upon phagocytosis are recognized as essential source of cellular ROS for inflammasome activation.^44,46^ During ROS-dependent inflammasome activation, elevated intracellular ROS allow dissociation of the NLRP-ligand (thioredoxin-interacting protein (TXNIP)) from the ROS-sensitive TXNIP–thioredoxin complex, which specifically bind with the leucine-rich repeat domain of NLRP3 to trigger inflammasome activation. In the channel model of NLRP3 inflammasome activation, the rapid K+ efflux facilitated by activation of P2X7 ATP-gated ion channel not only serve as requirement crucial for inflammasome activation but also produce low levels of intracellular ROS,^47^ suggesting an inflammasome–dependent positive feedback for sustained ROS production.^45^ These findings proposed potential mechanisms engaging cellular stress recognition and ROS production for subsequent NLRP3 inflammasome activation.^48^

### Inflammasome and pyroptosis

Pyroptosis, a form of programmed necrosis associated with release of cellular contents and pro-inflammatory cytokines, have essential roles in mediating protective innate immune response to combat invading pathogens and microbial infections.^49^ Hallmark events include (1) inflammasome priming, (2) NLRP3 inflammasome assembly and activation, (3) cleavage of gasdermin D (GSDMD) and pore formation as well as (4) pro-inflammatory molecules secretion (Fig. 1). Ample evidences detected pyroptosis activities and elevated IL-18/IL-1β levels in neutrophils observed in LPS- and cecal ligation and puncture (CLP)-induced sepsis models.^50^ Serum PCR array performed on clinical sepsis patients also proved the presence of an altered inflammasome-related gene profile, featured with a greater magnitude of altered genes and higher intensity of gene expression disturbance compared to normal controls. Expression levels for genes such as NLRP3, NLRC4, TLR5, NOD, IL-1β, and IL-18 showed intricate interconnection that could eventually lead to a robust inflammasome gene profile, while in sepsis non-survivors, a higher magnitude of the same gene modulation pattern were observed, revealing its clinical relevance with sepsis severity.^51^

The NLRP3 inflammasome activation event required at least two activating signals for triggering distinct processes (Fig. 1).^52^ Initially, pathogenic-derived molecules recognized by pattern-recognition receptors (PRRs) triggered nuclear translocation of NF-κB and transcription of pro-IL-1β and pro-IL-18. Studies using synthetic LPS and TLR4 activators were potent to induce apoptosis-associated speck-like protein (ASC) pyroptosome formation, illustrating their indispensable role for NLRP3 priming.^53^ Under LPS stimulation, a strong NF-κB-dependent NLRP3 mRNA expression was observed in macrophages in wild-type mouse, while a dose-dependent reduction of NLRP3 protein was observed in cells lacking TLR4 or double deficient in myeloid differentiation factor 88 (MyD88) and TRIF. Together with the generally low expression of NLRP3 levels observed in inactive immune cell lines,^54^ NF-κB signaling is suggested as the crucial priming event that guarantee inflammasome to respond accurately to stimuli and prevent inappropriate NLRP3 activation.^53^ However, recent study showed that mitochondrial ROS induce priming by directly leading to deubiquitination of NLRP3 inflammasome but independent of protein synthesis, suggesting a non-transcription priming mechanism for NLRP3 deubiquitination.^55^

**Fig. 2:**
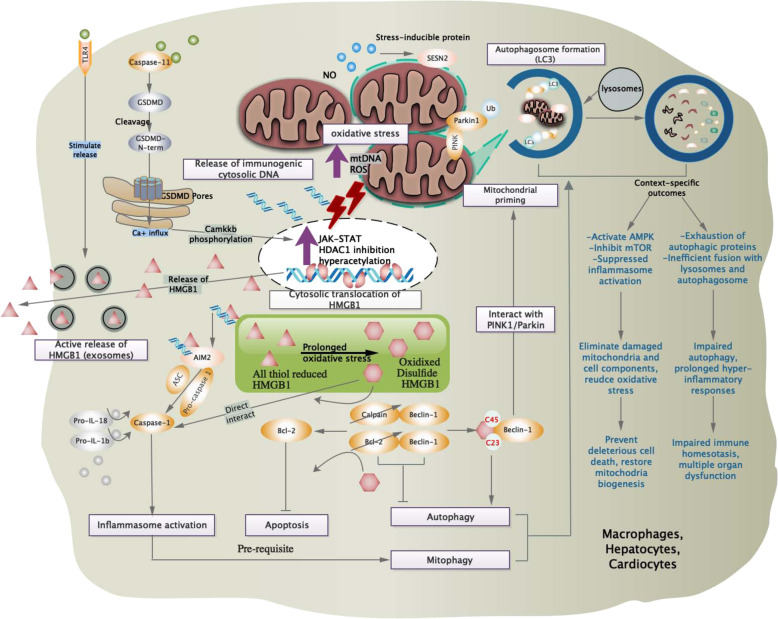
Stress signaling and cell homeostasis. Intracellularly, oxidative stress-mediated redox state is pivotal to maintain host cell survival and homeostasis via cross-talks with inflammasome, cell death pathways, and stress-responsive proteins. Mitochondria-derived ROS and mtDNA provide stimuli for upregulation of JAK-STAT pathway and inhibition of HDAC1, which is required for hyperacetylation and cytosolic translocation of HMBG1. In the presence of pathogenic stimuli, substantial release of all thiol-reduced HMGB1 by exosomes serve as inflammatory mediators that marked the prelude of sepsis. This redox form interacts with AIM2 and dsDNA to initiate inflammasome activation and caspase-1-mediated responses, which serve as pre-requisite for autophagy/mitophagy induction via beclin1-mediated pathways. Besides, prolonged oxidative stress oxidize HMGB1 into the disulfide form, which is potent to displace Bcl2 from its association with beclin1, allowing autophagy initiation and removal of hazardous oxidative stress stimulus. Interaction of beclin-1 with PINK/Parkin further facilitate mitochondria priming and autophagosome formation, which subsequently interact with lysosome to induce mitophagy. Autophagy and mitophagy are essential mechanisms to regulate innate immune response and prevent stress-induced injuries. During systemic inflammation context (sepsis), failure to initiate protective autophagy/mitophagy might result in profound cell stress signaling and deleterious pro-inflammatory cell deaths. HDAC1 histone deacetylase 1, Bcl-2 B cell lymphoma-2, GSDMD gasdermin D, SESN2 sestrin 2, PINK PTEN-induced putative kinase 1, LC3 microtubule-associated protein 1A/1B-light chain 3

Yet, priming signals provided by NF-κB activators were insufficient for inflammasome activation. In the absence of established NLRP3 activators, enhanced NLRP3 expression were inadequate for caspase-1 activation, revealing the requirement of a crucial inducing agent for NLRP3 inflammasome assembly.^53^ Experimentally, NLRP3 inflammasome activation was mediated by a diverse group of endogenous or exogenous agonists including DAMPS, extracellular ATP, pore-forming toxins (nigericin), or biochemical crystals (silica and alum).^56–58^ For instance, apoptosis inhibitor of macrophage 2 (AIM2) specifically recognizes cytosolic dsDNA and initiates inflammasome activation (Fig. 2).^59^ Extracellular ATP found in transfected models activates P2X7 receptor and NLRP3 signaling in an autocrine manner (Fig. 1),^58^ while significant switch of metabolism status (i.e., elevated saturated fatty acids, absence of ketogenesis) during sepsis converge with inflammasome signaling (Fig. 1) and was associated with increased lethality.^59^ Mechanically, these secondary signal activators function to increase membrane permeability for potassium efflux and subsequent downstream binding of NEK7 with NLRP3, therefore regulating NLRP3 oligomerization, inflammasome assembly, and subsequent catalytic cleavage of pro-caspases (Fig. 1).^60,61^

Some bacterial-specific toxin also interacts with host cells to exhibit NLRP3 activating properties. During *Staphylococcus aureus* infection, massive potassium efflux induced by potassium channel-forming toxins such as α-toxin was potent in activating inflammasome.^62^ Besides, mitochondria dysfunction during apoptosis or programmed cell death might result in significant reduction in negative potential within mitochondria, thus triggering release of oxidized mtDNA and cardiolipin to cytosol. Both molecules bound and induce NLRP3 inflammasome activation licensed with IL-1β production (Fig. 1).^63,64^

Recently, a TLR4-independent mechanism for inflammasome activation was found in intracellular Gram-negative bacterial infection. Intracellular LPS transfected by *Escherichia coli*,^65^ *Salmonella typhimurium*,^66^ and *Legionella pneumophila*^67^ were sensed and respond via a caspase-11 non-canonical inflammasome mechanism. Intracellular LPS trigger pyroptosis by directly binding to the recruitment domains (CARD) of pro-caspase-11, which subsequently underwent recruitment, oligomerization, and activation of pro-caspase-11 (Fig. 1).^68^ Caspase-11-dependent pyroptosis is a critical event for endotoxin shock in mice, representing an innate sensing mechanism. Analogously, similar pyroptotic cell death mediated by homologous caspase-4/5 were observed in human monocytic cell lines.^69^ To promote cytokine production, activated caspase-11 require NLRP3/ASC complex formation in a cell-intrinsic manner, independent of danger signals or pro-inflammatory cytokines released by adjacent cells. In this process, caspase-11 is rather upstream of NLRP3 activation and the caspase-11-mediated potassium efflux was a necessary intrinsic event for triggering non-canonical NLRP3 inflammasome activation.^60,70^ Noteworthy, the LPS-activated caspase-11 catalytically cleave GSDMD to release the intramolecular inhibition on the gasdermin-N domain but have no effect on pro-IL-1β and pro-IL-18 activation.^71^

Cleavage of GSDMD by active caspase-1/4/5/11 release functional active gasdermin-N domains that contain liposome-leakage-inducing and intrinsic pore-forming properties, subsequently forming membrane pores that facilitate active release of inflammatory cytokines (IL-18 and IL-1β) and intracellular components.^72,73^ Pore-forming properties of the gasdermin family contributed to the distinctive molecular and structural mechanism in pyroptosis. Unlike the explosive-like membrane blebbing and cell swelling that resulted from MLKL selective diffusion of ions during necroptosis, GSDMD-N pore facilitate non-selective ion diffusion without increasing osmolality.^74^

### Mitochondria dysfunction, inflammasome activation, and cell death

It was proven that engagement of specific NLRP3 inflammasome activators could amplify mitochondria destabilization, leading to pyroptotic cell death with enhanced loss of plasma membrane integrity and release of intracellular proteins (DAMP) via a caspase-1-dependent mechanism,^75^ while excessive ROS resulting from impaired electron transport chain, Ca^2+^ overload, or attenuation of endogenous anti-oxidants also triggered cell death modalities, such as apoptosis and autophagy.^76,77^ During mitochondria damage, the highly expressed mtROS promote apoptosis via triggering opening of mitochondrial permeability transition pores (mPTPs),^78^ while in an animal model of acute peritonitis, inhibition of mPTPs was sufficient to reduce sepsis-induced myocardial dysfunction.^79^

Induction of these ROS-mediated events partially relied on inflammasome activities, then interplay with cellular processes that would exacerbate to cell death. Physiological events leading to calcium influx not only provide calcium signaling required for regulation of NLRP3 inflammasome assembly but also initiate robust inflammasome activation by inducing the intermediate step—mitochondria destabilization.^80^ Therefore, calcium mobilization that lead to Ca^2+^ overload in mitochondria damage play a vital role in NLRP3 inflammasome activation.^81^ In the process of apoptosis, free oxidized mtDNA and cardiolipin released to cytosol also serves as a potent ligand for NLRP3 inflammasome activation (Fig. 1).^63^ During sepsis, activated caspase-1 interact with molecular events in precipitating mitochondria dysfunction, such as mitochondria ROS production, perturbation of membrane permeability, and fragmentation of mitochondria network, thus exacerbating apoptosis featured with pro-inflammatory responses.^75^ While demonstrating the role of a serum pro-inflammatory factor (S100A12) on NLRP3 inflammasome activation, the resulting apoptotic events orchestrated by elevated intracellular oxidative stress was suggested for pathogenesis of sepsis-induced acute respiratory distress syndrome.^82^ Likewise, anti-apoptotic proteins were mentioned to be effective in attenuating NLRP3 inflammasome responses.^42^

The types of mitochondria dysfunction might have consequences on the inflammatory nature of the ongoing cell death, regulated by formulation of apoptosome or inflammasome in a context-dependent manner.^83^ Cleaved caspase-1 by NLRP3 inflammasome could engage with multiple pathways in parallel to trigger mitochondrial damage that result in either pyroptosis or apoptosis. Mechanically, caspase-1 inactivates mitophagy process via catalytic cleavage of the key mitophagy regulator Parkin, thus allowing accumulation of dysfunctional mitochondria that augment pyroptosis.^75^ This additional caspase-dependent event corresponds to the inactivation of pro-survival and homeostatic pathways previously observed during apoptosis.

### Necroptosis

Different from the caspase-dependent type apoptosis, molecularly controlled forms of necrosis play predominant role in pathogenesis of cell death in sepsis. With defined signaling transduction pathways, necroptosis showed additional physiological significance and perform distinctly from typical necrosis. Via interacting with death receptors and various cytoplasmic protein kinases, necroptosis also drives inflammation and immunogenic events for various diseases.^84,85^ Unlike necrosis that often lead to irreversible pathological damages, necroptosis has been utilized by organism to obtain appropriate levels of cellular activity and embryonic development.^86^

Necroptosis was first notified as a TNF-induced type of necrosis and later confirmed as kinase-dependent process after the successful inhibition by RIPK1-inhibitor (necrostatin).^87^ Unlike caspase-8-regulated apoptosis, necroptosis was specifically regulated by signal transduction proteins known as RIPK1 and RIPK3, both of which function as complex of membrane-associated proteins.^88,89^ Ligation of stimuli induce interaction between coupled proteins and kinases that trigger formation of necroptosis-initiating cytosolic complexes, so as to respond with relevant cell death/survival outcome via inducing signal transduction. Aside from the established TNF/TNFR1-induced necroptosis, stimuli such as immune death signals (FASL/TRAIL),^90^ bacterial and cellular stress signals (LPS/TLR4, poly(I:C)/TLR3)^91,92^ as well as type-I/II IFNs (viral RNA/PKR or autocrine loop for sustained RIPK3 activation)^93,94^ were capable of inducing necrosome formation via stimulation of death receptors. TLRs (TLR3, TLR4) that signals through a RHIM-domain-containing protein (TRIF, DAI) could allow interaction of RIPK1 and RIPK3 and lead to necroptosis.^95,96^ Besides, recruitment of RIPK1 to Fas in the absence of inhibitor of apoptosis (IAP) trigger transformation of cytosolic complex I to complex II, which lead to assembly of necrosome and necroptosis.^97^ Conversely, activation of cytosolic complex I was known to trigger pro-inflammatory signaling and promote cell survival by NF-κB and MAPK activation.^98^

Though mechanisms determining apoptosis, necroptosis, or cell survival remains unclear, genetic studies revealed that FADD or caspase-8 deficiency could sensitize cells to undergo RIPK3-mediated necroptosis, thus triggering embryonic lethality and inflammation.^99,100^ Similarly, sufficient levels of catalytically active RIPK3 is required to induce necroptosis and suppress apoptosis.^101^ In clinical sepsis, RIPK3 levels were significantly elevated at various time points with positive correlation to SOFA score. Regarding its relationship with clinical outcome, lower levels of TRAIL, a potent inducer of apoptosis, is associated with increased levels of RIPK3, while RIPK3 levels is positively associated with higher incidence of organ dysfunction and septic shock, suggesting the pathological impacts of RIPK3-mediated pathways.^102^ In analog, in vivo sepsis studies have also proven the participation of RIPK3 necroptosis during organ deterioration.^103^

Unlike the irrelevant physiological roles between apoptosis and necroptosis, necroptosis and pyroptosis collaborate to amplify inflammatory signals and aggravate tissue injuries, both playing pivotal roles in the progress of sepsis. Though both processes were activated by a similar pool of stimuli signals, intracellular signals were transduced via distinct signaling pathways and target to their respective death regulator proteins—RIPK3 or GSDMD. In the effective phase, release of intracellular component varied between the type of programmed cell death—DAMPs such as high mobility group box protein 1 (HMGB1) released during necroptosis while pro-inflammatory cytokines IL-1β and IL-18 during pyroptosis. In view of the potential synergistic impact on sepsis-induced injuries, one study aimed to investigate the protective effect on double deletion of RIPK3 and GSDMD. Double blockage of necroptosis and pyroptosis by deletion of RIPK3/GSDMD or MLKL/GSDMD showed accumulated protective effects against septic shock, systemic blood clotting, and multi-organ injuries. Both RIPK3 and GSDMD perform lytic cell death that collaborate to amplify necro-inflammation and tissue factor (TF) release in macrophages and endothelial cells, resulting in massive tissue injuries.^104^

In neonatal polymicrobial sepsis mice, systemic and pulmonary inflammation were ameliorated with improved survival after injection of RIPK1 inhibitor (necrostatin-1). Decreased expression of local IL-6, IL-1β, and IL-18 as well as neutrophil chemoattractant mRNA were also observed,^105^ while deletion of RIPK3 confer complete protection against SIRS, with reduced amount of circulating DAMPs and cytokines.^106^ Similar protective effects were confirmed in CLP-induced sepsis models without negative effect on apoptosis or NK-kB activation, thus indicating the therapeutic potential by targeting necroptosis process.^107^ Having noted that evidence of complex II formation for necroptosis in tissue samples is hard to demonstrate, the above results had attempted to prove the involvement of necroptosis in sepsis-induced lung injury and presented the protective effect of RIPK inhibition on sepsis.

Recently demonstrated in in vivo study using LPS-challenged human kidney tubular epithelial cells, enhanced RIPK3 expression subsequently promote oxidative stress and mitochondrial dysfunction via upregulation of NADPH oxidase-4 and downregulation of mitochondrial complex I and III. These activated components are important evidences associated with sepsis-induced acute kidney injury (AKI). In this study, RIPK3-mediated responses work independently with RIPK1 and MLKL and were untypical to the necroptosis process. Together with observation of mitochondrial depolarization in in vitro study, these findings have shed insights into RIPK3’s unique role in regulating mitochondrial function during sepsis-induced AKI.^108^

### Autophagy and mitophagy

Autophagy and mitophagy are conserved processes essential for cell homeostasis and initiated under various stress conditions. Both function to facilitate intracellular degradation of dysfunctional mitochondria, damaged cytosolic organelles, and invading microorganisms, therefore mitigating the extent and severity of cellular-induced inflammatory responses.^109^ Autophagy might have alleviated inflammasome activity via multiple aspects, such as elimination of damaged mitochondria, removal of cytoplasmic HMGB1, and activated inflammasomes.^110^ Involvement of caspase-1-dependent activities in mitochondria damages have already been discussed in the previous section. Notably, ROS production by mitochondria subsequently oxidized HMGB1 released from apoptotic cells, resulting in neutralization of immunogenic activity and fail to activate innate immune cells (Fig. 2). Though reduction of oxidized HMGB1 by thioredoxin is sufficient to maintain the reduced form, such reaction is low in efficiency during sepsis.^111^

In contrary to the pathological role of serum HMGB1 in amplifying systemic inflammation, intracellular HMGB1 shows cross-talk with cell homeostasis and exert protective effect under specific circumstances. As for hepatocytes and macrophages, cytosolic HMGB1 is capable to prevent deleterious cell death from endotoxemia by mediating autophagy and mitophagy (Fig. 2).^112^ Stress stimuli that enhance ROS could promote nucleocytoplasmic shuttling of HMGB1, where it directly interacts with the autophagy protein beclin-1 by displacing Bcl-2, thus resulting in formation of autophagy initiation complexes and removal of hazardous oxidative stress stimulus (Fig. 2).^113^ The various redox states of intracellular HMGB1 have contributed to its important regulatory role for AIM inflammasome activation. All thiol reduced form of intracellular HMGB1 showed highest affinity when binding with AIM2, which subsequently initiate inflammasome signaling during redox stress (Fig. 2). Initiation of inflammasome pathway serves as an important prerequisite for stimulating protective autophagy and mitophagy for cell survival.^114^

The disulfide bridge formed between HMGB1 cysteines and beclin-1 is an essential conformation structure required for sustained autophagy.^115^ Furthermore, HMGB1 controls the checkpoint process that proceed to autophagy, via preventing the calpain-mediated cleavage of autophagic regulator beclin-1 and ATG5 during inflammation.^116,117^ Though autophagy level was downregulated proportionally according to severity of sepsis condition, injection of cell-permeable TAT-beclin-1 successfully restore mitochondrial biogenesis and preserve sepsis cardiac function via PINK1/Parkin and AMPK/ULK1 signaling. Initially, PINK1 protein on outer mitochondria membrane recruit and activate Parkin that builds ubiquitin chains on damaged mitochondria, facilitating its binding to LC3 on the autophagosome to induce mitophagy (Fig. 2). By interacting with Parkin, Beclin-1 is potent to support PINK1/Parkin-mediated mitophagy via localization of mitochondria-associated membrane for directing ER–mitochondria tethering and inducing the formation of autophagosome precursors for mitophagy.^118,119^

As an immunomodulatory molecule, endogenous nitric oxide (NO) have been identified as a negative regulator for NLRP3 inflammasome activation via stabilization of mitochondria in macrophages.^120^ In response to stress-mediated cellular dysfunction, NO is potent to balance inflammasome responses and promote survival via inducing autophagy. Through elimination of mtROS and mtDNA, the ROS-mediated autophagy helps suppress NLRP inflammasome hyperactivation and maintain stability of mitochondrial function.^110,121^ In recent study, a novel negative regulator for macrophage pyroptosis during sepsis was identified. As an anti-oxidant enzyme responsible for repairing oxidative lipid damage, glutathione peroxidase 4 (GPX4) catalyzes reduction process of phospholipid hydroperoxide to inhibit lipid peroxidation. Besides, GPX4 exhibit coordinated role for oxidative stress, inflammasome activation, and pyroptotic cell death. During sepsis, GPX4 was proposed to inhibit phospholipase C gamma 1-mediated GSDMD activity and caspase-dependent events, therefore reducing excessive macrophage pyroptosis.^122^

However, excessive NO production due to TXNIP deficiency increase sensitivity to lethal endotoxic shock. After LPS treatment, TXNIP expression was observed to decrease dramatically and then gradually restored, which coincidentally accompany with a significant increase of inducible NO synthase (iNOS) expression. With multiple biological functions on oxidative stress, cell proliferation, and inflammation, these might have contributed to the distinct susceptibility phenotype observed in TXNIP-deficient status. Previously noted, TXNIP exhibit a protective negative regulatory role on NO production, induction of iNOS expression as well as on NF-κB activation, while in TXNIP-deficient mice, increased sepsis susceptibility proceed despite reduced IL-1β processing by S-nitrosylation of NLRP3. These results emphasize the involvement of other crucial roles of TXNIP on inflammatory response. Finally, it was suggested that TXNIP participated with the regulation of NO production via the NF-κB/iNOS pathway.^123^

However, inhibition or upregulation of autophagy process depends to the context-specific activated pathways and confronted stress. In models of severe sepsis, autophagy is downregulated and insufficient to counteract the NLRP3-induced negative outcomes due to exhaustion of autophagic proteins (Fig. 2).^118^ In endotoxemia models with severe abdominal infection, inefficient fusion of autophagosomes with lysosomes result in impaired autophagy (Fig. 2). Inefficient clearance of autophagic vacuoles and bacterial products remain source of stress stimuli, leading to hyper-inflammatory response via induction of cGAS-STING pathway.^124^ Besides, the stress-inducible proteins SESNs (sestrins) suppress prolonged NLRP3 inflammasome activation via inducing mitophagy by a two-phase cooperative mechanism (Fig. 2). First, SESNs facilitate priming of damaged mitochondria by inducing aggregation of SQSTM1 to the Lys 63-ubiquitinated (U) mitochondria. Coupled with maintained levels of ULK1, a specific autophagic machinery was finally triggered for degradation of primed mitochondria.^125^ This SESN-mediated mitophagy provides a previously unknown mechanism aside from regulating antioxidant expression and lowering ROS levels.^126,127^

### Warburg effect and metabolic reprogramming

Pyruvate kinase M2 (PKM2), a kinase that interact with hypoxia-inducible factor 1α (HIF-1α), was capable to mediate HMGB1 release via exerting inhibitory signal for histone deacetylases (HDACs), providing a novel mechanism for metabolic control on inflammation.^128^ Previously, LPS was shown potent in elevating the transcriptional regulator HIF-1α, via a TLR4-depedent fashion. With evidences of massive inflammatory cytokines (TNF-α, IL-1, IL-4, IL-6, and IL-12) associated with HIF-1α expression, HIF-1α was proven as a critical determinator for sepsis phenotype.^129^ Similar signs of altered metabolism were later observed in activated innate immune cells (dendrite cells and macrophages).^130,131^ During sepsis, presence of hypoxia, inflammatory, or infectious signals prevented HIF-1α from degradation. Accumulated levels of HIF-1α specifically interact with PKM2 to promote targeted gene expression (aerobic glycolysis-related genes) in a positive feedback so as to mimic Warburg effect (Fig. 1).^132,133^ Warburg effect was first observed in cancer cells,^134^ characterized with upregulated levels of glycolysis and lactate products even under normoxic conditions. Such effect is essential to provide biosynthetic requirements conducive for cell proliferation rather than efficient ATP production.^132,135^ During aerobic glycolysis, excessive production of PKM2-mediated lactates inhibit HDAC activity and result in elevated acetylated levels of HMGB1 comparable to that stimulated by LPS and HDAC inhibitors.^128^ Acetylated HMGB1 was then translocated to the cytosol and subsequently released into extracellular space. This reprogrammed mechanism parallel with previous studies that have also demonstrated the pivotal role of HDAC in regulating mobilization of acetylated HMGB1 during liver ischemia and reperfusion (I/R) injury.^136^

More importantly, PKM2-dependent glycolysis promote NLRP and AIM inflammasome activation via PKR autophosphorylation (Fig. 1).^137^ PKR (also termed as EIF2AK2), a double-stranded RNA-dependent protein kinase, was previously known as an intracellular viral RNA sensor and recently proven to be primed by endogenous cellular and metabolic stress signals. Lactate-induced PKR phosphorylation and activation promote release of IL-1β, IL-18, and HMGB1 in macrophages, indicating the regulatory role of PKM2-PKR complex in inflammasome activation (Fig. 2). Via reducing PKR phosphorylation and caspase-1 activity, both PKM2 inhibitors (shikonin) and PKR inhibitors (C16) were capable to protect mice from lethal sepsis. Autophosphorylated PKR could physically interact with specific inflammasome components (NLRP3, NLRP1, NLRC4, AIM2) and subsequently trigger corresponding events of inflammasome activation. Therefore, PKR was indicated as an integral component between innate immune responses and stress stimuli, while inhibitors of PKR were suggested as novel therapeutic targets to counteract inflammation.^138^ In contrast, other studies suggested that PKR is not required for inflammasome activation and might even inhibit inflammasome activity to avoid initial priming during innate immune response.^139,140^

### Pink1-Park2 pathway: mitophagy and immunomodulatory role

Participation of Pink1-Park2 pathway in maintenance of mitochondrial quality control via mitophagy has been widely studied,^141^ while a novel Pink1-Park2 protective neuro-immune pathway during sepsis was recently mentioned. In genetic depletion of genes encoding for Pink and Park2, a subsequent decrease in neurotransmitter dopamine was accompanied with increase of late sepsis mediator—HMGB1, via mechanism of HIF-1α-dependent anaerobic glycolysis and NLRP3 inflammasome activation. This finding has proposed the involvement of Pink1 and Park2 neuro-immune pathway in regulating dopamine release and HMGB1 secretion, which exacerbate sepsis severity via activating NLRP3 inflammasome.^142^

In consistent with previous observation of PKM2-HIF-1α metabolic pathway that promote AIM and NLRP3 inflammasome activation, the HIF-1α-mediated anaerobic glycolysis upregulated after depletion of neurotransmitter-mediated immune responses showed association with sepsis lethality. Together with the pro-inflammatory role of PKM2-HIF-1α on LPS-activated macrophages,^133^ HIF-1α-mediated immune-metabolic dysfunction was emphasized as a mechanism for lethal sepsis. Indeed, recent studies shed lights on the protective role of Pink1-parkin pathway by mitophagy in AKI.^143,144^ Featured with accelerated elimination of damaged mitochondria, Pink1- and Park2-mediated mitophagy prevented cell apoptosis and tissue damages through reducing mitochondrial ROS and subsequent NLRP3 inflammasome activation. These findings further support the regulatory role of a novel Pink1-Park2 pathway on immune-metabolism during sepsis, providing rationale basis for cross-interaction of host defense system with intrinsic cellular responses.

### Coagulation cascades

**Fig. 3:**
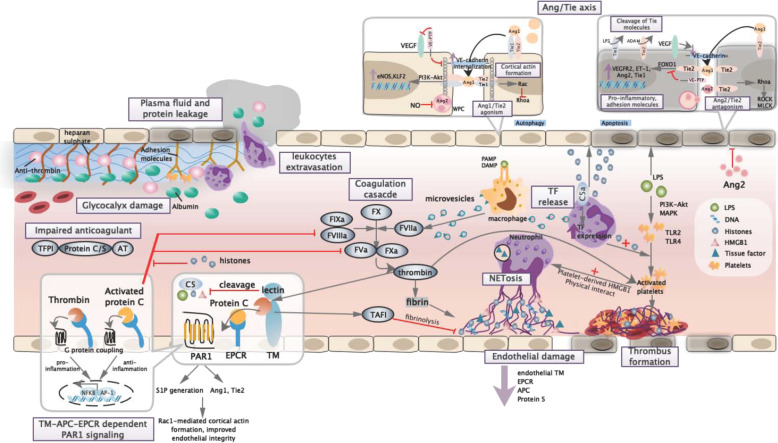
Endothelial barrier and coagulation cascades. Lined by membrane-binding proteoglycans and glycosaminoglycan side chains, dearrangement of endothelial glycocalyx result in loss of anti-thrombogenicity and exposure of adhesion molecules, which allow leukocyte adhesion, platelet recruitment, and thrombus formation. Increased vascular permeability trigger leukocyte extravasation, plasma protein leakage, and tissue edema. Under immunogenic stimuli, procoagulant tissue factors were substantially expressed in the form of microvesicles by macrophages and neutrophils, which triggered extrinsic coagulation cascade and cleavage of prothrombin. Neutrophils and platelets work reciprocally to regulate innate immune response and bacterial clearance via NETosis and platelet-mediated responses. During sepsis, NET-derived histones HMGB1 and cfDNA abundantly found serve as procoagulants that accelerate thrombus formation, while activated platelets and RBC recruitment result in thrombocytopenia and platelet-rich thrombus. In order to monitor coagulation dynamics, thrombin interacts with endothelial-bound TM to exhibit profound anti-coagulant and anti-inflammatory effects. In the presence of APC-EPCR, TM/thrombin interact with coagulation–fibrolysis system to act as negative feedback loop. APC–EPCR interaction also allow switching of PAR-1 signaling to anti-inflammatory pattern and strengthened endothelial integrity via activities mediated by S1P and Ang/Tie axis. Besides, TM/thrombin directly inactivate procoagulant alarmins to further restrict immunocoagulation. During severe infection, dysregulation of Ang/Tie axis were associated with weakened vascular stability, which include inhibition of junctional protein, destabilization of cortical actin, and increased vascular adhesion and permeability. Finally, extensive formation of inflammatory thrombus and activation of coagulation cascades result in microcirculatory dysfunction and organ injury. TM thrombomodulin, PAR-1 protease activated receptor 1, EPCR endothelial protein C receptor, S1P sphingosine-1 phosphate, TFPI tissue factor pathway inhibitor, AT antithrombin, TAFI thrombin-activatable fibrinolysis inhibitor, Rac RAS-related C3 botulinum toxin substrate, RhoA ras homolog family member A, Ang1 angiopoietin 1, Ang2 angiopoietin 2, VEGF vascular endothelial growth factor, VE-PTP vascular endothelial protein tyrosine phosphatase, WPB Weibel–Palade body, MLCK myosin light chain kinase, ROCK Rho-associated kinase, ADAM disintegrin and metalloproteinase domain-containing protein

Disseminated intravascular coagulation (DIC) is a life-threatening syndrome with excessive activation of intravenous coagulation cascades, exhaustion of anti-coagulants, and suppressed fibrinolysis. In sepsis, approximately 35% of the severe cases showed complication of DIC.^145^ Incidence of DIC was significantly increased in late phase sepsis correlated with irreversible septic shock and organ dysfunction.^146^ Mortality rate in septic DIC patients is almost twofolds higher compared to those without DIC.^147^ Systemic activation of the coagulation and inflammation cascades have central role in the pathogenesis of DIC. During initial phase, extensive expression of inflammatory cytokine-mediated TF was known as the principal mechanism of DIC (Fig. 3). In both endotoxemia and sepsis models, coagulation largely depends on the levels of TF expressed on macrophages and monocytes,^148,149^ while damaged endothelial cells and neutrophils might provide alternative sources of TF.^150^

Insufficient balance of TF-dependent coagulation events by tissue factor pathway inhibitor (TFPI) in early phase supported the impaired physiological function of anti-coagulants recognized during sepsis.^151^ Systemic inflammation amplified coagulation cascade via inflammasome mechanism and synergistic effect with other innate immune components. Besides, bacterial-derived products and DAMPs (NETs, HMGB1, cfDNAs, and histones) participated and accelerated development of DIC via multiple aspects (Fig. 3).^152–154^ Minimal levels of TF derived from perivascular cells due to increased vascular permeability have also contributed to the coagulopathy.^155,156^ Exposure of TF attract interaction with the FVII and FX that activate both coagulation cascades,^157^ which gradually generate prothrombinase complex that covert prothrombin to thrombin and induce fibrin formation for platelet activation. Conversely, thrombin and other coagulation factors exhibit arrays of pro-inflammatory activities via cleavage of protease-activated receptors (PARs), forming a positive feedback loop that augments inflammation and coagulation (Fig. 3).

### Endothelial barrier dysfunction

Endothelial dysfunction is one of the hallmark features of sepsis. Stability in endothelial cell contacts allows systemic blood flow, supply of essential biological molecules, and effective immune defense to take place. Integrity of endothelial barrier maintained by intracellular junction molecules regulates anti-coagulant and anti-inflammatory properties of endothelial lining. In response to pathogen-derived molecules and cell stress signals, secretion of cytokines and chemokines recruit neutrophils to localized infection sites for phagocytosis and oxidative killing to take place, while upregulation of endothelial adhesion molecules and loosening of vascular tight junction favor cell migration to targeted tissues. However, increased endothelial permeability further enhance microvascular leakage that trigger vascular hypotension, tissue edema, organ failure, and shock (Fig. 3).

Endothelial cell barrier destabilization was activated via alteration in intracellular junction molecule expression and dynamics of cytoskeleton contraction. Such functional changes were exclusively regulated by endothelial-specific receptors known as Tie receptors (including Tie1 and Tie2). Signaling was diversely mediated by two secreted Angiopoietin (Ang) family proteins—Ang1 as agonist while Ang2 as context-dependent agonist/antagonist.^158^ Investigation of circulating biomarkers reveal a correlation between plasma Ang2 levels and severity of acute respiratory distress syndrome (ARDS), while in sepsis studies, Ang2 serves as a biomarker for sepsis severity and is related to sepsis progression.^159,160^

Under stable vasculature, Angs (Ang1 and Ang2) interact with Tie receptors and enhance formation of Tie1/Tie2 heterodimer, which result in Tie2 trafficking to cell–cell junctions (Fig. 3).^161^ Tie2 then trigger cortical actin formation and upregulate pathways related to anti-adhesion and anti-inflammatory properties of endothelial cells, therefore maintaining vascular stability. The Ang/Tie2 pathway poses broad impacts on vascular remodeling, inflammation, and cell survival of endothelial cells. Ang/Tie2 activation induce the expression of KLF2 via PI3K/Akt pathway and counteract with the vascular endothelial growth factor (VEGF)-mediated vascular permeability, while elevation of intracellular NO by endothelial NOS (eNOS) expression is potent to inhibit exocytosis of Ang2 from Weibel–Palade bodies (WPB) found in endothelial cell cytoplasm (Fig. 3).^162,163^

## Essential cells and signaling molecules

### High mobility group box protein 1

As an evolutionarily conserved DNA-binding protein presenting with high electrophoretic mobility, HMGB1 was abundantly found in the nucleus and initially known to play essential roles in maintaining genome homeostasis and cell survival.^164,165^ During sterile injuries and infections, extracellular HMGB1 is able to alert innate immune response and regulate inflammation by acting as an endogenous prototypical DAMP.^166^ Later, HMGB1 is proven as a late-phase inflammatory mediator that drives endotoxin lethality in sepsis. Elevation of HMGB1 was detected considerably later than secretion of acute-phase cytokines (such as TNF and IL-1) and reach a persistent plateau at 16–32 h in LPS-induced endotoxemia models and sepsis patients.^165,167^ Besides, remarkable elevation of HMGB1 is observed in severe sepsis patients and correlate to disease progression.^168,169^

In resting cells, HMGB1 was found anchored to nucleus and stabilized by chromatins.^165^ HMGB1 could be released extracellularly either by active post-translational modification that facilitate nucleocytoplasmic translocation or by passive diffusion from damaged lytic cells. HMGB1 functions were determined by the types of post-translational modification, sites of activation, sources of cell types, and redox states.^111,170^ Various studies have demonstrated HMGB1’s role in regulating inflammasome activation, autophagy, cell survival, coagulation, and innate immunity by using sepsis model,^171^ suggesting the potentially multifaceted roles of HMGB1 in sepsis pathogenesis. In response to exogenous and endogenous danger signals, activated immune (macrophages, monocytes, and neutrophils) and non-immune (hepatocytes) cells release HMGB1 through multiple signaling pathways. HMGB1 released extracellularly act as alarmins to promote arrays of inflammatory cytokines with a delayed and biphasic pattern.^172^ Moreover, HMGB1 release was shown as the downstream mediator of inflammasome activation.^173^ Recent finding revealed the essential role of HMGB1 in delivering extracellular LPS required for non-canonical caspase-11 inflammasome activation and pyroptotic cell death (Fig. 1).^174^ Functioning as a DAMP, researchers also argued that HMGB1 secretion induced by type I IFN and TNF signal in necrotic cells might be linked with necroptotic pathway and required for necroptosis-induced inflammation.^175–177^

Biological effects of HMGB1 largely depend on the levels of nucleocytoplasmic shuttling and accumulation. Post-translational modification such as acetylation, phosphorylation, and methylation of multiple amino acid residues within nuclear localization sequences (NLSs) could result in HMGB1 translocation.^172^ HMGB1 acetylation is specifically regulated by histone deacetylase (HDAC) and histone acetylase (HAT).^178^ In several sepsis studies, both type I-IFN- and type II-IFN-mediated JAK-STAT were identified as the upstream promoting signal required for HMGB1 hyperacetylation.^179–181^ JAK-STAT signaling have been suggested to be involved with HMGB1 expression, hyperacetylation, and translocation in various activated immune cells (Fig. 1).^179,182^ As observed in ischemia-reperfusion injured-hepatocytes, the JAK/STAT-activated IRF physically interact with nuclear histone acetyltransferase enzyme p300 so as to regulate acetylation status of HMGB1,^183^ whereas a similar JAK-STAT-IRF-1 signaling was responsible for LPS-induced HMGB1 acetylation and essential for cytoplasmic accumulation.^179^

Phosphorylation of HMGB1 serve as another post-translational mechanism for active nucleocytoplasmic translocation. Concomitant phosphorylation on serine located at NLS1 and NLS2 sites might reduce its proximity and alter the three-dimensional structure required for KAP-a1 binding (a nuclear cargo-binding protein), therefore allowing cytoplasmic accumulation of HMGB1 and preventing relocation to nucleus.^184^ Calcium-mediated signaling might be the underlined mechanism for HMGB1 phosphorylation. Calcium/calmodulin-dependent protein kinase (CaMK) IV-dependent HMGB1 serine phosphorylation was known to mediate HMGB1 shuttling in LPS-stimulated macrophages,^185^ while in ischemic liver tissues, HMGB1 secretion adopt a regulated process facilitated by redox stress (ROS), which later induce CaMK signaling involved with CaMK IV and Camkk β.^186^ Moreover, CaMK I was shown to be involved in the release of HMGB1 via enhancing IFN-β signaling by a process of indirect phosphorylation of IRF3.^187^ As Camkk β catalyzes on its substrates (i.e., CaMK I and CaMK IV) and that CaMK signaling is upstream of HDAC inhibition, redox activation of CaMKs might trigger initial release of HMGB1 during sepsis.

Active release of HMGB1 from hepatocytes were identified as the major source of pro-inflammatory systemic HMGB1 in endotoxemia and CLP sepsis.^174^ The translocation of HMGB1 required co-activation of both TLR4 and caspase11/GSDMD signaling (Fig. 2).^188^ In line, increased TLR4 activation and intracellular uptake of LPS induce GSDMD cleavage via direct activation of caspase-11 found in cytosolic compartment of macrophages. However, signs of pyroptosis and lytic cell death was not observed in these models.^189^ Instead, activated GSDMD promote HMGB1 translocation and release into exosomes. Different from membrane GSDMD pores formed during pyroptosis, accumulation of cleaved GSDMD on ER facilitate free calcium leakage and promote calcium-dependent signaling by phosphorylation of Camkk β.^188^ Camkk β then act as upstream regulator of HDAC inhibition, which further lead to hyperacetylation and nucleocytoplasmic translocation of HMGB1 (Fig. 2),^186^ while extracellular release of HMGB1 requires receptor-specific TLR4 signals but independent of caspase-11 and GSDMD activation.

Previously, interaction of receptor for advanced glycation end products (RAGE) and HMGB1 in macrophages was shown to trigger pyroptosome formation, caspase-1 activation, and pyroptosis after endocytosis.^190^ Endocytosis of HMGB1 undergo a cascade of molecular events that result in release of cathepsin B from ruptured lysosomes, a key event for pyroptosome formation (Fig. 1). Cathepsin B is potent to directly interact with NLRP3 at the ER levels, resulting in pyroptosome formation and pro-caspase-1 activation.^191^ As demonstrated in sepsis model, neutrophil extracellular trap (NET)-derived HMGB1 was indicated as the distinct source for caspase-1-dependent macrophage pyroptosis associated with augmented pro-inflammatory activities.^192^

A recent study has unraveled the critical role of circulating HMGB1 in mediating lethal sepsis. It clearly illustrated the complete molecular mechanism of LPS-HMGB1 complex in initiating caspase-11-dependent pyroptosis.^174^ In response to PAMPs such as LPS and Poly(I:C), substantial level of HMGB1 was released by hepatocytes. Circulating HMGB1 promptly bound with LPS to form HMGB1-LPS complex and mediate translocation of extracellular LPS via RAGE. RAGE-mediated internalization of HMGB1-LPS into endo-lysosome serves as the critical step for cytosol delivery of LPS. The acidic pH provided by lysosomes subsequently enhance direct permeabilization of lysosome phospholipid bilayer by HMGB1, inducing transfer of LPS to cytosolic caspase and subsequent caspase-11-dependent pyroptosis for LPS lethality.

Though other studies have argued that HMGB1 itself could trigger ASC-dependent and caspase-11-independent pyroptosis,^190^ it was believed that the redox status of HMGB1 have contributed to these inconsistent observations. The three cysteines located in the A box and B box of HMGB1 was modified under different redox states.^193^ The all-thiol fully reduced form referred to the presence of reduced SH thiol groups at all cysteines. During early sepsis, HMGB1 is released in the all-thiol fully reduced form.^194^ It forms heterocomplex with CXCL12 that binds to receptor CXCR4 to elicit chemotactic activity but not TLR4-dependent cytokine release.^195^ In this redox state, HMGB1 serve as endotoxin delivery protein that bind and internalizes LPS into lysosome, an intermediate step required for LPS cytosol release and activation of non-canonical caspase-11-dependent pyroptosis (Fig. 2).^174^

As inflammation proceed and oxidative products accumulates, C23 and C45 in A box are close enough to be oxidized and form disulfide bond, while the third cysteine (C106) in B box remain reduced.^196^ This disulfide form of HMGB1 is capable in mediating massive inflammatory responses such as TNF-α release and NF-κB signaling by allowing binding of reduced C106 with TLR4-MD2.^196,197^ HMGB1 is also capable to interact on TLR2 and RAGE to elicit cytokine-like activity. The pro-inflammatory type of HMGB1 was predominantly detected at weeks 4–8 in sepsis survivors.^194^ Studies have claimed that pro-inflammatory effect largely depend on the B box due to its much lower dissociation rate with TLR4.^198^ Besides, disulfide form of HMGB1 alone could prime NLRP3 inflammasome activation for stimulating excessive cytokine production.^199^

Under massive oxidative stress, release of ROS from mitochondria abrogate both chemotactic and cytokine stimulating activity of HMGB1 and transited into the irreversible fully oxidized HMGB1. This inactive form of HMGB1 were found at weeks 8–12 after inflammation or during hepatic regeneration,^194^ therefore preventing from excessive inflammatory damages. Fully oxidized HMGB1 also function as an important element for induction of immunological tolerance in apoptotic cells. ROS production resulted from caspase cleavage of mitochondria substrate p75 neutralizes the stimulatory activity of HMGB1, thus avoiding the dangerous immune response elicited by the highly inflammatory HMGB1 once released from apoptotic cells.^200,201^ Redox modifications of HMGB1 might serve as a dynamic biological switch for its inflammatory activity in response to different magnitudes of oxidative stress.

### Interactions of complements with hemostasis and pathogens

Initially, endothelial barrier damage by endotoxins and inflammatory cytokines result in exposure of endothelial collagen fiber and TF, which trigger platelet aggregation that activate thrombin release and fibrin formation. Once platelets are activated, the released serine/threonine-dependent protein kinase could phosphorylate residues of the C3d region of C3. Phosphorylation of other C3 fragments could prevent the cleavage degradation of C3b, resulting in persisted complement activation.^202^

Complement components reversely feedback to promote coagulation during activation. C5a induce the expression of TF and plasminogen-activator inhibitor 1 on various cell types, while shedding heparin sulfate on vascular endothelial.^203,204^ Similarly, membrane attack complex (MAC) induce the expression of TF in attached cell and adhesion molecule in endothelial cell lines.^35,205^ In platelets, MAC facilitate the expression of binding site and catalytic surface for the prothrombinase complex.^206^ Other complement components such as C1q and C3a enhance procoagulant activity and induce aggregation of platelets.^207,208^ An appropriate homeostasis between complement and coagulation system guaranteed the full performance of protective inflammatory response and pathogen clearance.

Pathogens could adapt and exploit host defense via interrupting with specific checkpoints of complement system, thus impairing the normal coupling between complements and TLR signaling pathway. Etiology-dependent mechanism has evolved as a decisive factor on the initiation of complement cascade, either via classical or MBL pathway. Certain microbial species have adopted specific mechanisms to evade complement attack. *Porphyromonas gingivalis* enzymatically cleaves C5 to generate high concentration of C5a for C5aR1 activation on neutrophils while coincidently detected by TLR2. This enhanced C5aR1–TLR2 cross-talk would lead to degradation of downstream signaling adapter MyD88, which is essential for bacterial clearance. After evasion of host immune surveillance, dysbiotic communities depends on host inflammatory products for metabolism and survival. The C5aR1–TLR2 cross-talk alternatively trigger activation of PI3K signaling that inhibit phagocytosis and induce inflammatory responses so as to provide a nutritionally favorable micro-environment that result in dysbiosis and disease development.^209^

Opsonized pathogens by C3b might end up in distinct intracellular outcomes depending on the activated signaling pathways. Certain non-enveloped bacteria opsonized extracellularly by C3 fragments escaped from phagosomes could still be detected by cytosolic C3 and trigger mitochondrial antiviral signaling, which robustly induce pro-inflammatory cytokines via upregulation of several transcription factor (NF-κB, AP-1, IRF-3, and IRF-5) and promote viral degradation.^210^ Conversely, uptake of C3-coated *Francisella tularensis* by C3R consequently activate Lyn kinase and AKT signaling, which in turn upregulate MAPK phosphatase-1 and inhibit MAPK-dependent pro-inflammatory responses downstream of TLR2, allowing persistent intracellular bacterial survival.^209^

### Extracellular histones

Under various clinical scenarios (sepsis, trauma, cancer, and ischemia), substantial elevation of extracellular histones were detected after significant cell death.^211–213^ In the form of circulating nucleosomes or NETs, extracellular histones are established DAMPs that activate immune cells via TLR or NLR signaling pathways.^214,215^ Neutrophil-derived NETs function to trap pathogenic microbes and exhibit robust bactericidal effect.^216^ Within NETs, networks of DNA fibers and histones have provided scaffold for platelet aggregation, cell localization (neutrophils, erythrocytes), and activation, which further enhance formation of red blood cell-rich micro-thrombi.^152^ In comparison to DNA fibers, histones appear to have a more significant effect on clot formation by improving the mechanical stability in thrombi.^217^

However, excessive release of histones from dying cells and NETS during sepsis might be accompanied with cell cytotoxicity.^218^ Sub-lethal dose of histone causes early death in mice, presented with pathological features mimicking sepsis, such as neutrophil accumulation, vacuolated endothelial cells, and formation of macro- and micro-thrombi.^219^ Similarly, significant elevation of plasma histones were observed during sepsis deteriorating stages, accompanied with sepsis-related cytokines and highly correlated with mortality, organ dysfunction, and thrombocytopenia.^220,221^

In histone-mediated kidney and liver injuries, main pathological features include extensive cellular damage, hemostatic imbalance, and amplification of inflammatory responses.^212,222^ Both TLR2 and TLR4 were shown to have prominent role in histone-mediated cell toxicity, mediating signal transduction for MyD88, NF-κB, and MAPK pathways. Interestingly, genetic ablation of either TLR2 or TLR4 alone did not abrogate cytokine production, suggesting the requirement of both TLRs.^222^ In course of septic cardiomyopathy, both TLR3 and TLR9 were linked with histone-induced damage of cardiomyocytes. As demonstrated in TLR3 and TLR9 knockout animals, significant reduction in plasma levels of C5a, histones as well as cytokines in heart tissues were observed, confirming the detrimental roles of TLR3 and TLR9.^223^ Unexpectedly, study regarding the molecular events in complement-mediated lung injury identified a link between complement interactions with extracellular histones. Ligation of C5a with its receptors (C5aR and C5L2) triggered release of histones (H3 and H4) from activated neutrophils, which result in acute lung injury featured with intense inflammation, polymorphonuclear neutrophil accumulation, and alveolar epithelial cells damage (Fig. 3).^224^

Though numerous studies have established mechanism responsible for increased histone cytotoxicity, molecular events of extracellular histone-mediated endothelial dysfunction remains unclear. Recently, a dose-dependent mechanism of autophagy and apoptosis mediated by extracellular histones was confirmed in cultured human endothelial cell lines. Such responses were shown to be mediated via sestrin2/AMPK/ULK1-mTOR and Akt/mTOR pathways.^225^ In consistent with previous results, the type of histone-mediated cell death, either autophagy or apoptosis, is determined by concentration of extracellular histones (Fig. 3).^226^ Initially, low concentration of histones direct cells to autophagy via upregulation of sestrin2/AMPK/ULK1-mTOR pathways and decrease in Akt activation, while subsequent inactivation of mTOR and dephosphorylation of p70S6K (mTOR downstream target) also contribute to autophagy. As histone concentration increases, extracellular histones immediately induce a p53-dependent upregulation of Bax and result in apoptosis, which in turn inhibit the expression of autophagic protein Bcl-2. Involvement of both autophagic and apoptotic pathways in the dose-dependent mechanism of histone-related cytotoxicity have provided novel potential targets for therapeutic strategies.

Recently, a TLR9-dependent mechanism was identified for histone-mediated inflammation observed in I/R-injured Kupffer cells.^227^ Further investigation proposed novel role of histones in propagating I/R liver injury. Extracellular histones were hypothesized to directly interact and activate TLR9-mediated ROS generation, which further triggered NLRP3 inflammasome activation and recruitment of additional cell types, thus driving innate immune response that exacerbates I/R injury.^228^ In addition, stimulating histones in TLR-KO models have observed a dose-dependent increase in inflammasome activation within TLR2 and TLR4 KO cells but not in TLR9 KO cells, suggesting a significant role of TLR9 in histone-mediated inflammation.^228^ Other reports documented that TLR9 activation by histones was attributed to contamination,^229,230^ as TLR9 originally function for sensing intracellular DNA but not histones.^231^ This suggested that DNA-binding histones might have acted in conjunction to facilitate DNA-mediated TLR9 response.

Exclusively, extracellular histone promote thrombi generation via platelet-dependent mechanisms (Fig. 3)^232^ by directly modulating the clotting properties of platelet-induced polyphosphate (polyP), allowing its induction of thrombi generation with histones.^233^ Moreover, histone enhance procoagulant phenotype of platelets by upregulation of P-selectin, phosphatidylserine (PS), and FV/Va, partially in a TLR2- and TLR4-dependent mechanism.^232^ The enhanced platelet aggregation might contribute to formation of platelet-rich thrombi in sepsis. Direct histone–platelet interaction was also suggested as the potential mechanism for rapid profound thrombocytopenia.^234^ Histone preferentially bind with platelet plasma membrane via a charge-dependent manner. Subsequent induction of calcium influx promote activation of αIIbβ3 on platelets, together with specific binding to a platelet receptor or fibrinogen facilitated by histone, were responsible for the formation of large platelet aggregates that cause profound thrombocytopenia.

### Platelets: crucial intermediators between inflammation and hemostasis

Upon extensive thrombus formation, platelets are sequentially activated and aggregated to the site of impaired vascular integrity, providing a perfect scaffold for coagulation cascades to take place.^235^ While during sepsis, rapid thrombocytopenia was associated with decreased platelets adhesion, increased bacterial load, and enhanced inflammatory cytokines, which further modulate coagulation–fibrinolysis system and impaired hemostasis process.^236–238^ In clinical sepsis, recruitment of LPS-induced platelets were detected in lung and liver microvascular thrombosis,^239–241^ in a TLR4-dependent manner. Supported with evidence of direct LPS receptor TLR4 expressed on platelet, a reciprocal relationship in LPS-induced platelet response was proposed.^242^ Discrepancy in antigenicity of LPS from different bacterial strains further determined its function on platelet responses. It was suggested that rough form of LPS directly interact with platelet, independent of soluble CD14 required by smooth LPS, might have participated in LPS-induced platelet activation.^243^

Several studies have investigated on the underlying signaling pathway during platelet activation. Akt, the downstream effector of PI3K pathway, as well as small GTPase (JNK, ERK, p38) implicated in the MAPK pathway, were observed to be activated in LPS-induced platelets in in vivo models.^244,245^ Elevated ROS was proven to be a required element for platelet activation, as administration of antioxidants and inhibition of relevant signaling pathway significantly reduce LPS-induced platelet function.^244,245^ To conclude, LPS-enhanced platelet activation is capable to initiate TLR4/PI3k/Akt-ERK1/2/PLA2 pathway that involves TXA2 and ROS generation. TXA2, the downstream product of PLA2 activation, is necessary for effective LPS action on platelets, while ROS is required to modulate LPS-TLR4 signaling at different levels.^246^ In stimulated platelets, PLA2 was activated in response to actions of Akt, thus stimulating a second wave of platelet aggregation. In addition, activation of the MAPK-ERK1/2 pathway is involved with platelet stimulation and ROS release under regulation of PI3K/Akt, in consistent with the broad biological effects of PI3K pathway reviewed previously.^247^

HMGB1 was also critical for the pathogenesis of coagulation abnormalities observed in trauma and hemorrhagic shock.^248,249^ In in vivo models of injury-induced thrombosis, elevated HMGB1 levels within thrombi was indicated as platelet-derived and had presumably contributed to inflammation and organ failure. By evaluating the molecular mechanism of HMGB1-driven platelet activation during thrombosis, critical role of HMGB1 was again established.^250^ Characterized with platelets activation, dense granule secretion, aggregation, and thrombus formation, HMGB1 potentially modulate the pro-thrombotic phenotype of platelets via complex formation of MyD88/GC and cGMP-dependent protein kinase (cGKI) pathway. It has been proven that platelets store and express HMGB1 on cell surface upon activation.^251,252^ Platelet-derived HMGB1 specifically interact with TLR4 to induce a MyD88-dependent GC recruitment toward platelet plasma membrane.^253^ Elevation of intracellular cGMP immediately induce downstream activation of cGKI, an essential signal required for TLR4/HMGB1-mediated thrombus formation and platelet aggregation.^250^

In interaction with adhesion protein such as von Willebrand factor (vWF), activation of platelet integrin affinity was enhanced via MAPK/ERK pathway in a cGKI mechanism, while MAPK-mediated activation of platelet integrin αIIbβ3 require signaling via platelet GPIb-IX receptor.^254^ In addition, insufficient degradation of vWF due to ADAMTS13 deficiency might have enhanced thrombosis in sepsis and mimic certain clinical presentation of thrombotic thrombocytopenia purpura, supported by evidence of low levels of ADAMTS13 and association with DIC severity.^255,256^

### Coagulation: cross-talks between inflammasome, ROS, and TF

Bacterial-derived products trigger delivery of LPS into cytosol of host cells, which might be responsible for occurrence of DIC.^257^ Systemic activation of coagulation was recently revealed to be involved with caspase-11-dependent inflammasome activation.^258^ Demonstrated by monocytes and macrophages, activation of the LPS cytosolic receptor caspase-11 and formation of GSDMD pores initiate coagulation cascade via PS exposure (Fig. 1). The GSDMD pore-mediated Ca^2+^ influx promote exposure of PS on the outer leaflet of plasma membrane via activation of transmembrane protein 16F (TMEM16F), a calcium-dependent phospholipid scramblase. PS externalization allow activation of TF and assembly of cofactor–protease complexes during coagulation cascade. To date, molecules such as HMGB1 and bacterial outer membrane vesicles that are required for localization of LPS in cytosol might have taken advantage on this immune-thrombotic mechanism in triggering systemic coagulation during sepsis.^259,260^

In light of the advanced understanding of the role of caspase-11 and GSDMD during endotoxin-induced coagulation, this provided evidence for a previously undetermined relationship between type-I IFN signaling and intrinsic coagulation cascades. As coagulation process is intended for host to prevent dissemination of pathogenic bacteria,^261^ infection-induced type-I IFN serve as a crucial intermediator between innate immune response and coagulation.^262^ Induced by Gram-negative bacteria, LPS-mediated type-I IFN signaling amplify HMGB1 extracellular release via direct modification on acetylation levels at the nuclear location sequences. Extracellular HMGB1 has already proven to increase pro-coagulant activity of TF via promoting externalization of PS to the outer plasma membrane. While the corresponding caspase-11 activation converge as upstream signals for GSDMD-dependent PS exposure, other mechanism might have contributed to coagulation cascades such as thiol-disulfide exchange, NETosis, platelet activation, and disruption of endothelial Tie2 axis.^263–266^

TMEM173 (STING), a classic innate immune sensor that stimulate the expression of IFN in response to DAMP (i.e., cytosolic DNA),^267^ was identified to be involved with coagulation via triggering ER stress-induced GSDMD activation. TMEM173 were significantly activated during infection and inter-related with disease severity.^268,269^ Implication of excessive TMEM173 activation in sepsis pathogenesis have been widely discussed.^27,269–271^ TMEM173 interact with a predominant calcium channel ITPR1 to promote ER calcium efflux required for caspase-1/11/8-induced GSDMD cleavage and activation. The ER stress-mediated GSDMD pore formation then initiate pyroptosis and subsequent release of TF.^272^ Prolonged innate immune activation by TMEM173 had consistently promoted inflammasome coagulation process with the presence of GSDMD pores.

With the observation of extensive TF-driven coagulation events particularly in pyroptotic macrophages, other study agreed on an inflammasome-dependent mechanism that had supported a central role of TF in pathogenesis of pyroptotic cell death. In LPS-induced models, massive thrombosis and systemic coagulation triggered by increased TF activity were associated with inflammasome overactivation. Pyroptosis was mentioned as the trigger for lethal DIC not only via promoting TF-positive microvesicles formed by cell membrane fragment of pyroptotic macrophages but also triggering cell rupture required for extracellular TF release.^273^

Aside from post-transcriptional activation of TF via caspase-11-dependent PS exposure, stimulation of TF prothrombotic properties might require certain redox partners.^263^ Recently, generation of procoagulant microparticles (TF-bearing microparticles) in response to extracellular ATP signal was concluded to signal via caspase-1 pathway under regulation by Trx/TrxR system.^274^ The Trx/TrxR system was known to be associated with redox modification.^275^ Though experimental study had proposed a protective role of Trx,^276^ clinical studies have consistently observed elevation of plasma Trx levels in sepsis non-survivors, which is positively correlated with cytokines levels and lethal coagulation.^275,277,278^ Activated by P2X7 receptors, TrxR-dependent release of Trx and subsequent extracellular thiol-disulfide exchange result in progressive oxidation of extracellular Trx, which is analogous to the PDI-dependent TF activation mechanism. Intracellular depletion of Trx further lead to TXNIP-dependent inflammasome activation for IL-1β production and caspase-1 required for extracellular actin-dependent MP generation. Activation of caspase-1/calpain cysteine cascades then result in filamin degradation and release of TF for raft-dependent incorporation into PS-rich MPs. Besides, distinct composition such as TF and P-selectin levels have also contributed to the prothrombotic properties of these microparticles.^279,280^

### Neutrophils, platelets, and coagulation

Several studies have demonstrated the crucial role of neutrophils in promoting thrombogenesis.^281,282^ Expression of TF in neutrophils was considered as an initiating event in coagulation cascade.^283–285^ In sepsis model, detection of TF-positive granulocytes and established regulatory mechanism for TF gene expression have provided evidences suggestive of in situ synthesis of TF by neutrophils.^286^ However, incapable to detect TF mRNA expression in TF-positive granuocytes,^286^ studies argue that neutrophil TF was not produced in situ but rather up-taken from cell vehicles such as monocyte-derived microparticles.^287^ In contrast, anaphylatoxin C5a was able to mediate in situ TF production by neutrophils via upregulation of TF gene,^288^ providing a convincing mechanism for de novo TF production by neutrophils (Fig. 3). Such C5a-dependent manner was also observed in other clinical situations, such as ARDS and end-stage renal disease.^283,285^

During sepsis, enhanced NET formation was observed subsequent to TLR4-mediated platelet binding to adherent neutrophils.^289^ TLR4 on platelet serve as a threshold switch for signaling pathogen stimuli to a neutrophil-mediated bacterial trapping mechanism. Comprised with DNA fibers and histones, NET is suffice to provide scaffold for fibrin deposition, platelet entrapment, and stabilization of thrombus essential for hemostasis (Fig. 3).^152,290^ However, overwhelmed platelet activation and DAMP released from NET compounds risk causing massive tissue and endothelial damages.^241^ As demonstrated in sepsis-induced hepatic injured tissues, NET-induced detachment of sinusoidal endothelial cells allows platelets to enter the space of Disse, where platelets bind with collagen-3 to initiate extravasated platelet aggregation, a thrombotic event leading to irreversible deterioration of hepatic function.

Platelet-derived HMGB1 was previously shown to be critical initiators of NET formation via interacting with RAGE.^291^ Extracellular delivery of platelet-derived HMGB1 to neutrophil serves as a causative signal to prime NET formation via promoting autophagy. Accumulation of TF and HMGB1 in acidified autophagosomes (LC3B-coated vacuoles) facilitate its delivery and localization to cytosolic NETs, which subsequently stimulate NETosis, release of TF, and initiation of coagulation cascade. Existence of an autophagic machinery during release of TF-bearing NETs suggested a novel secretory mechanism for membrane- or cytosolic-bound proteins toward NETs. This also emphasized that inflammatory mediators such as HMGB1 serve as crucial stimuli for post-transcriptional regulation of TF. More importantly, recruitment of neutrophils to damaged endothelial cells and release of TF-bearing NETs result in thrombus formation that could persistently activate inflammation responses via PAR signaling pathways.^292^

### Thrombomodulin (TM), protein C, and endothelial protein C receptor (EPCR)

Thrombomodulin (TM) is an endothelial transmembrane glycoprotein that possess a central modulatory role in the natural anticoagulant system. Acting as a cofactor of thrombin, it reversibly formed TM–thrombin complex that blocks thrombin’s binding properties with pro-coagulant substrates, such as fibrinogen, protease-activated receptors, and coagulation factors V and VIII.^293^ Besides, binding of thrombin by TM inactivates its capacity for PAR-1 signaling and the downstream pro-inflammatory responses (Fig. 3).^294^ As the downstream target of PAR-1 signaling, decreased induction of ERK1/2 is also responsible for suppressed mitogenic effect of thrombin, via induction of NOS3 and generation of NO by TM–thrombin.^295^ TM–thrombin cross-talk with fibrinolytic cascade by accelerating activation of TAFI (thrombin-activatable fibrinolysis inhibitor), which render fibrin to be tolerant to plasminogen binding (Fig. 3).^296^ In consequence, TAFI effectively degrade pro-inflammatory mediators including bradykinin and anaphylatoxin complements (C3a and C5a).^297,298^ Independent of the anticoagulant property elicited by activation of protein C, the lectin-like domain of TM neutralize pro-inflammatory activity of thrombin via suppression of leukocyte adhesion,^299^ interference with complement activation,^300,301^ inactivation of HMGB1, histones,^302^ and bacterial endotoxin (Fig. 3).^303^

In transgenic mice expressing lectin-like domain-deleted TM, increased susceptibility to endotoxin shock is featured with enhanced expression of intercellular adhesion molecule and vascular cell adhesion molecule-mediated leukocytes. Recombinant lectin-domain of TM (rTMD1) was proven sufficient to restore normal leukocytes adhesion via suppressing TNF-α-induced ERK phosphorylation.^299^ Various studies had revealed molecular mechanism of the anti-inflammatory effect of TMD1. TMD1 structure within the thrombin–TM complex could specifically degrade HMGB1 to a less pro-inflammatory form that block its interaction with RAGE, while rTMD1 interfere with LPS-CD14 binding and inhibit subsequent LPS-induced inflammation via suppressing the MAPK and NF-κB signaling pathway as well as iNOS expression in macrophages.^303,304^ In animal models, recombinant TM significantly reduce LPS-induced sepsis mortality characterized with reduced levels of TNF-α and inflammatory cell infiltration in the lungs and livers.^303,305^

TM-dependent protein C activation augment the above-mentioned anti-coagulant and anti-inflammatory effects.^306^ Enhanced by cofactor protein S located on activated platelets and endothelial surface, the APC-PS complex proteolytically degrade coagulation factor Va and VIIIa to obtain anti-coagulant effect (Fig. 3).^307^ Efficacy of protein C activation could be further facilitated after interaction with EPCR.^308^ The resulting APC-ECPR complex downregulates NF-κB signaling and inflammatory cytokine production, via a PAR-1-dependent mechanism (Fig. 3).^309,310^

As a potent biomarker for reflecting severity of endothelial damages, downexpression of endothelial TM was vastly observed in the dermal microvasculature of patients with meningococcal sepsis.^311^ Similarly, the proteolytic degraded form namely “plasma-soluble TM” was distinctly observed in severe sepsis patients. Soluble TM was markedly elevated in septic patients with organ failure and in consistent with the serial changes of endothelial activation markers during the course of sepsis.^312,313^ Analogously, levels of soluble EPCR was shown correlated with poor outcomes in sepsis patients.^314^ Unexpectedly, formation of thrombin that generally result in upregulation of EPCR might also contribute to EPCR shedding during endotoxin shock.^315,316^ The elevated pro-inflammatory cytokines TNF-α and IL-1β during sepsis have enhanced EPCR shedding via activation of the MAPK pathway.^317^ In consistent, administration of APC showed limited protective effect in septic mice with low expression of EPCR, therefore emphasizing the requirement of EPCR- and PAR-1-dependent effect for APC’s efficacy in reversing sepsis-related mortality.^318^

Interaction with TM switch substrate specificity of thrombin from pro-coagulant (fibrin) to anti-coagulant (protein C).^319^ Following activation of protein-C, TM was potent to augment anti-inflammatory effects via APC-dependent mechanisms (Fig. 3).^306^ By interfering with NF-κB and AP-1 pathways, APC inhibited endotoxin-induced TNF-α production in human monocytes,^320^ which is parallel to results observed in endotoxemic mice. Recombinant APC was also reported to be therapeutically beneficial in severe sepsis patients presented with overt DIC.^321^ It is evident that APC interact with EPCR to produce enhanced anti-coagulant responses upon excessive thrombin generation.

Interestingly, thrombin exhibit bifunctional roles on endothelial barrier integrity via inducing dual effects of PAR-1 signaling in a thrombin concentration-dependent mechanism (Fig. 3). Both PAR-1 agonist peptide and low concentration of thrombin induce barrier-protective effect comparable to that observed in elevated APC condition. It was proposed that cross-talks of receptor systems have contributed to a balanced regulation of endothelial function in a dual-chamber system via PAR-1 signaling.^322^ Certainly, it is now clear that APC-EPCR complex interrupt with the effector response of thrombin by recoupling the PAR-1 to bind with alternative members of the G protein families responsible for multiple intracellular signaling pathways.^323^ In such response, occupancy of EPCR by APC induce dissociation of caveolin-1 from EPCR and recouple PAR-1 to the alternative Gi protein, thus preventing activation of the pro-inflammatory RhoA pathway related to cytoskeleton remodeling and exhibit an endothelial barrier protective effect.^324^ In addition, activated PAR-1 also improve endothelial function via inducing sphingosine-1-phosphate (S1P) generation and transactivation of S1P receptor type 1 (S1P1). Such response improves endothelial barrier stabilization via activating the Rac1-mediated cortical actin formation.^325^ Enhanced endothelial barrier mediates anti-inflammatory effect via reducing leukocyte extravasation to sites of inflammation (Fig. 3).^322,326^ EPCR-APC-dependent activation of PAR-1 signaling also promote barrier stabilization via activation of Ang/Tie2 system (Fig. 3).^327,328^ EPCR-dependent PAR-1 cleavage by low levels of thrombin increases the expression levels of Ang1 and Tie2 but downregulates Ang2, thus initiating a previously undefined protective mechanism by restoring endothelial integrity.^324^ Serving as potential strategies on endothelial barrier protection, both transactivation of S1P receptors and Ang1/Tie2 axis activation enhance barrier cell survival via the PI3K/Akt pathway.^326,329^

As a consequence of acute phase responses, increased plasma levels of C4bBP may account for the relative decrease in free protein S and reduced anti-coagulant activity observed in sepsis.^330^ In *E. coli-*infused baboons, inhibition of protein S by infusion of C4bBP result in a hypercoagulable state associated with enhanced lethality.^331^ Similar results were observed in condition of microvascular thrombotic disorder, such as hemolytic uremic syndrome.^332^ Inactivation of EPCR by neutralizing monoclonal antibody showed increased morbidity in *E. coli* baboon model while protein C administration protected against microvascular thrombotic response.^332^ Implicated from previous findings that interaction with cationic proteins potentially influence activities of TM–protein C system, extracellular histone, a late phase mediator for cell death and thrombin generation during sepsis, impair anti-coagulant activity via directly interacting with TM and protein C (Fig. 3). Additionally, co-factor activity of both soluble and endothelial TM were reduced.^333^ In view of this, preclinical trials using recombinant human form of TM (TM alfa) successfully restored equivalent anticoagulant effect. Clinically relevant concentration of TM alfa increased APC generation, leading to consequent cleavage of pro-coagulant histones and attenuated histone-induced endothelial cytotoxicity.^334^

### Sphingosine-1-phosphate

Sphingosine-1-phosphate (S1P) is a signaling lipid abundantly found in circulation and mainly bound to high-density lipoprotein (HDL)-associated apolipoprotein M and serum albumin.^335^ In PAR-1-activated endothelial cell, S1P is produced by phosphorylation of sphingosine by sphingosine kinase 1 (SphK1) and function to protect endothelial barrier integrity via enhancing cortical actin formation.^336^ In addition, S1P signaling recruits VE-cadherin for endothelial junction assembly via a Rac-1-dependent mechanism.^337^ In fact, S1P might have served as a bifunctional molecule. These contradicting functions were most likely due to the relative concentration and source of S1P production.^338,339^ In endotoxemia models, protein expression and enzymatic activity of SphK1 were observed to be elevated and responsible for hyper-inflammatory responses observed in macrophages.^340,341^ Recently, S1P was suggested as a DAMP involved with regulation of NLRP3 inflammasome activation in macrophages,^342^ while inhibition of SphK1 was shown to protect mice from sepsis-induced mortality. By suppressing NLRP3 inflammasome activation, this led to attenuated microvascular leakage via suppressing IL-1β-mediated adherens junction disassembly and endothelial cadherin internalization,^343^ though the involved signaling mechanism of NLRP3 inflammasome inhibition by SphK1 remains unknown. SphK1 was recently observed to regulate HMGB1 translocation by direct interaction with the CaMKII-δ. In sepsis-associated liver injury models, co-localization of SphK1 and CaMKII-δ in Kupffer cells provide signals for CaMKII-δ autophosphorylation. Activated CaMKII-δ serve as upstream regulator for HDAC4 phosphorylation, an inhibitory form of HDAC4 that favors hyperacetylation and translocation of HMGB1.^344^

In other studies, S1P signaling failed to exhibit endothelial protective effect over time due to the loss of endothelial responsiveness after prolonged exposure.^345^ Besides, the type of activating stimuli and S1P receptor coupled have determined whether a pro-inflammatory or barrier-enhancing feature would be exhibited by S1P. Still, S1P represents a potential therapeutic target with broad regulatory effect on endothelial barrier function and innate immune cells.^325^ In sepsis baboon models, plasma S1P was rapidly decreased within 6–8 h after *E. coli* injection.^346^ With particular significance for its regulatory role in endothelial barrier function, levels of serum S1P was dramatically decreased in end-stage severe sepsis patients, while similar changes were observed for the levels of S1P-binding molecules.^347–349^ Serum and plasma S1P levels were inversely correlated to disease severity in terms of septic shock incidence and higher SOFA scores.^350^

With ample evidences from several studies, it is rationale to claim that protective effect of PAR-1 signaling to be at least partially attributed to the anti-apoptotic effect of enhanced S1P-mediated responses.^322^ In early studies regarding endothelial function, APC is potent to inhibit staurosporine-induced apoptosis and modulate gene expression of the endothelial apoptotic pathway (Bcl-2, eNOS, and the IAP).^351,352^ Likewise, PAR-1 agonist had exhibited significant anti-apoptotic effect in human brain endothelium.^353^ As observed in embryonic stem cells, both Erk1/2 and PI3K/Akt signaling pathways were shown to be critical for the HDL/ApoM/S1P-mediated anti-apoptotic effect that promote stem cell survival.^354,355^ The EPCR-APC-mediated cell signaling was associated with cytoprotective and anti-inflammatory phenotype, featured with downregulation of vascular adhesion molecules, stabilization of endothelial barrier function, and inhibition of inflammatory and apoptotic signaling.^356^

### Cross-talks of STING with immune pathways

Based on the pivotal role of STING in modulating innate immune response in sepsis, studies have focused to identify key regulators for the STING pathway. By screening potential pharmacological kinase inhibitors for STING-mediated type I IFN responses, a novel regulatory target for STING-mediated innate response was proposed—anapestic lymphoma receptor tyrosine kinase (ALK). Previously known as a tumor-associated receptor tyrosine kinase, expression of ALK was relatively low in healthy individuals. While components of both ALK and STING pathway were abundantly expressed and upregulated in innate immune cells during clinical sepsis, such observation was in accordance with the elevated DNA levels from invading pathogens and host damaged cells specifically observed in poor outcome patients.^357,358^

Pharmacological inhibition or genetic lockout of either ALK or STING protect mice from lethal sepsis by restricting excessive STING-mediated innate recognition of bacterial DNA and subsequent pro-inflammatory cytokine expression (Fig. 1). It was proposed that ALK might have interacted with other receptor tyrosine kinase (RTK) on cell surface so as to mediate signal transduction that interfere with STING-initiated signaling pathways. Mechanistically, ALK/EGFR binding was shown to trigger AKT-dependent STING activation in innate immune cells and provided evidence for a novel signaling pathway that have contributed to sepsis pathogenesis and septic shock (Fig. 1).^359^ In summary, ALK-EGFR-AKT pathway was proven as a critical modulator of lethal STING-mediated innate immune responses. This novel role of ALK in modulating inflammatory signaling pathway has opened new avenue for development of therapeutic targeting drugs.

As a Food and Drug Administration (FDA)-approved second-generation ALK inhibitor, LDK378 exhibit STING-dependent anti-inflammatory effect in innate immune cells that rendered mice more resistant to lethal sepsis. Recent studies have demonstrated that LDK378 alleviate sepsis-induced pathological changes in heart, lung, and kidney. LDK378 improve micro- and macro-circulation via regulating STING-mediated cytokine release and microcirculatory dysfunction.^270^ However, some scientists argued that off-target effect of ALK inhibition might have contributed to the anti-inflammatory protection observed.

Recent work using LDK378 have identified novel function regarding host immune response. LDK378 was shown to interfere with LPS-mediated CCR2 upregulation that is required for recruitment of myeloid-derived suppressor cells (MDSCs) to peripheral lymph organs. Such effect was blocked via inhibiting the phosphorylation of p38 and subsequent G protein-coupled receptor kinase-2 in bone marrow MDSCs, which subsequently lead to CCR2 internalization and desensitization. Here LDK378 was proven to interact with p38-MAPK pathway in which the p38/GRK/CCR2 pathway plays pivotal role in chronic immunosuppression. During sepsis, high levels of circulating MDSCs abundantly found in the spleen, lymph nodes and bone marrows were correlated with increased nosocomial infection and poorer functional status at hospital discharge.^360^ MDSC is known as a type of suppressive innate immune cells that mainly function via secretion of ROS and NO, induction of T cell suppression, and selective polarization of Th-2 cells. By partially inhibiting recruitment of MSDC, LDK378 relieves the immunosuppression and inflammatory responses induced in lethal sepsis.

In the previous study, acquired mutation of STING was proven to disrupt ER calcium homeostasis and prime T cell to be hyper-responsive to TCR signaling-induced ER stress. This STING-mediated chronic elevation of ER stress was potent to prime T cell death by apoptosis.^361^ Expression of STING and GSDMD correlates with severity of disseminated intravascular coagulation and mortality in sepsis patients. Such STING-dependent systemic coagulation was independent from the classic STING-induced pathway that have already been mentioned in the previous section. Based on the multiple impacts on immune homeostasis, coagulation, and inflammation, STING pathway has been recently proposed as another potent therapeutic target for sepsis.

### Ang/Tie2 pathway and regulatory mechanism

Ang/Tie2 pathway was regulated and linked with a complexity of signaling pathways (Fig. 3). In the context of sepsis, stimuli of inflammatory cytokines, coagulants, or VEGF allow secretion of Ang2 from WPB.^162,362^ Ang2 itself serve as a rapidly acting regulator of the endothelium through an autocrine loop mechanism.^363^ In the presence of LPS or infection signals, ectodomain cleavage of Tie1 block Tie2 phosphorylation and contribute to loss of agonist property of Ang2 as well as weakened Ang1 activity.^158,161^ This suggest the indispensable role of Tie1 in Tie2 signaling. LPS-mediated Ang2 expression was found to be regulated by NADPH oxidase 2 (Nox2) via the IKKb/NF-κB and MAPK/AP-1 signaling pathways.^364^ The specific structure of Ang2 that prevent scattered Tie2 molecules from clustering to form multimers^365^ serve as the core mechanism in Ang2/Tie2 antagonism. In endothelial cells, Tie2 inactivation promotes nuclear translocation of FOXO1 and inhibition of KLF2, which enhance transcription of target genes, such as Ang2, VEGFR2, and ET-1,^366,367^ while increased expression of VEGFR2 activate downstream pathway such as eNOS, PI3K/Akt and Src, resulting in disruption of adhesion molecules (degradation of VE-cadherin and S-nitrosylation of beta-catenin).^368,369^

Reduction of Tie2 expression is another distinct feature observed in sepsis models and could further contribute to microvascular leakage. The VEGF-mediated PI3K/Akt pathway subsequently cross-interact with a p38 MAPK-dependent protease (ADAM) that is capable in cleaving Tie2 ectodomain into soluble Tie2 (sTie2).^370^ Likewise, changes in the levels of sTie2 were observed in critically ill sepsis patients.^371^ Under hypoxia condition, increased levels of VE-PTP was associated with Tie2 and act as a negative feedback mechanism by limiting the Tie2 downstream activity.^372^ In addition, decline Tie2 mRNA levels was known to be induced by decreased endothelial shear stress in a NF-κB-dependent manner.^373^

From gene transduction by recombinant virus to recombinant human antibodies targeting on Ang/Tie2 axis, several studies have proposed effective therapeutic strategies in restoring endothelial stability in sepsis murine models. Via augmenting Ang1, inhibiting Ang2, and promoting Tie2 activation, preclinical studies have demonstrated promising results in different sources of endothelial cells under various settings. Rh-Ang1 protein is capable to prevent pulmonary capillary leakage and preserve integrity of blood–brain barrier (BBB) after intravenous administration.^374,375^ Rosiglitazone, a PPAR-γ agonist that result in higher brain and plasma levels of Ang1 and lower Ang2/Ang1 ratios, is able to restore BBB integrity, achieve better neurocognitive outcomes, and improved survivals in malaria-infected mice.^376^

Considerable elevated levels of Ang2 in response to infectious signals makes it an ideal pharmaceutical target for restoring Tie2 signaling during sepsis. The anti-Ang2 antibody, ABTAA demonstrated a novel mechanism in stabilizing Ang/Tie2 axis during sepsis. It specifically targets on the deleterious Ang2 clusters by converting it into a high molecular complex that bind and act as agonist of Tie2.^377^ Tie2 activation protect sepsis progression by strengthening the endothelial glycocalyx, amelioration of cytokine storm, and vascular leakage in targeted organs.^377^ Other compounds such as rh-Ang1 variant (COMP-Ang1, MAT.Ang1), inhibitors of VE-PTP (AKB-9778), and synthetic Tie2 agonist (Vasculotide) directly modified the functional state of their reciprocal targets so as to preserve endothelial barrier integrity.^374,378–380^

Besides, Ang2 is capable in inducing RHoA activation via SHP-2-dependent dephosphorylation of RHoGAP (GTP activation protein), which activate downstream Rho kinase (ROCK).^381^ Such signaling contribute to cytoskeleton rearrangement (stress fiber assembly, cell contact destabilization) and endothelial junction breakdown (internalization of adhesion and tight junctions).^382^ Besides, increased secretion of thrombin and TNF-α during sepsis favors stress fiber assembly and cell contraction by RhoA-mediated activation of ROCK and myosin light chain kinase.^383^ As the downstream target of RhoA, ROCK-mediated regulation in vascular permeability was suggested to be a critical mechanism in sepsis-induced lung injuries.^384^

Actin-binding proteins (ABPs) are another group of regulating proteins involved in modulation of actin and myosin function during cytoskeleton remodeling.^385^ ABPs maintain endothelial stabilization by directly binding to actin/myosin and induce actin remodeling via polymerization, branching, and severing.^385^ Besides, ABPs cross-interact with complexity of intracellular signaling pathways via activation of small GTPases (Rac1, Rap1). Expression of NF-κB, MyD88, and p38 MAPK were also found to be involved in ABP regulatory signaling machinery.

## Intervention therapies in sepsis

Conventional management interventions such as early antibiotics, fluid resuscitation, and hemodynamic support by vasopressors have been effective for early sepsis resuscitation and significantly improve overall clinical outcomes. Yet, treatment targeting the underlying causes that had incited to devastating manifestation seems pivotal but remains unclear. Recognized as the host-mediated systemic inflammatory responses to infection, numerous clinical studies have observed evidences of immune cell activation and dysregulated host responses in severe sepsis patients that coincide with much of the established molecular mechanisms, together with the development of therapeutic monoclonal antibodies proven with safe profile in clinical sepsis patients. These provide strong rationales for further investigation on larger sepsis population.

Over the past two decades, multi-center randomized controlled trials (RCTs) have failed to obtain successful results using therapeutic strategies targeting on inhibition of specific components involved with inflammatory responses (i.e., IL-1, IL-6, TNF-α; Table 1) and coagulation cascades (Table 2). Discrepancy in sepsis clinical definition as well as multifaceted manifestations observed during clinical course have made such syndrome a more complicated clinical situation to investigate on. Besides, inclusion criteria of patients and observed clinical endpoints showed inconsistency between trials. Some of the measuring indicators seems inappropriate for clinical implication and incapable to address their full performance on clinical efficacy, which added up to the inherent limitation in these studies. Meta-analysis concluded low-quality levels of evidences from previous RCTs and subtle values for clinical translation.

**Table 1:** Comparison of therapeutic effects on biological agents targeted on sepsis-associated signaling pathways

Pharmacological target	Targeted mechanism	Year	Population	Study phase and design	Drug	Results	Conclusion	Source
Interleukin-1 receptor antagonist	Modifying the IL-1-mediated cytokine storm and inhibition of systemic inflammatory response syndrome in sepsis	1994	893	Phase III RCT	rhIL-1RA	No significant survival benefits among all sepsis patients	No effect among sepsis patients. However, a dose-related increase in survival time were observed among patients with organ failure and higher predicted risk of mortality	^402^
		2018	529	Re-analysis of a prior phase III study (1994)	rhIL-1RA	Patients with available plasma samples and clinical information were included (59%). Patients with initial plasma IL-1RA levels above empirical threshold (2071 pg/ml) showed reduced mortality after rhIL-1RA therapy	Heterogeneous effect of rhIL-1RA on sepsis mortality is potentially predictable by initial plasma IL-1RA level	^403^
		1997	696	Phase III RCT	Anakinra	All-cause mortality was reduced in the treatment group, but failed to achieve significant difference when analyzed on the basis of site of infection, infecting microorganism, presence of bacteremia, shock, organ dysfunction etc.	Therapeutic benefits are small and difficult to demonstrate in the sepsis population	^404^
		2016	43	Re-analysis of a prior phase III study (1997)	Anakinra	From the original cohort, 5.6% of sepsis patients were noted with MAS features. Subgroup patients with sepsis and concurrent HBD/DIC (features of MAS) is associated with significant improvement in survival	Anakinra provided survival benefits for sepsis patients with features of MAS	^405^
Recombinant human IL-7 (CYT107)	Interleukin-7 exhibit anti-apoptotic effect that is essential for lymphocyte proliferation and survival. CYT107, a glycosylated recombinant human IL-7, was previously administered in clinical trials to >390 patients and showed promising effect in increasing lymphocytes counts	2018	27	Phase II RCT	CYT107	CYT107 was effective in reversing sepsis-induced lymphopenia and caused a 3–4-fold increase in absolute lymphocyte counts and in circulating CD4+ and CD8+ T cells that persisted for weeks after drug administration	CYT107 reverse key pathogenic hallmark in sepsis and plays important role in sepsis-induced morbidity and mortality, without inducing cytokine storm or worsening ongoing inflammation	^406^
Anti-tumor necrosis factor-α fragment antibody	TNF-α is one of the principal initiators of the inflammatory cascade that underlies the pathology of sepsis, by stimulating the widespread release of downstream pro-inflammatory cytokines, such as IL-6 and IL-8	1996	141	RCT	Etanercept	TNFR:Fc fusion protein (Etanercept) does not reduce mortality and higher doses appear to be associated with increased mortality		^407^
		2001	1342	Phase III RCT	Lenercept	No effect on mortality reduction		^408^
		2013	70	Phase IIa RCT	AZD9773	Serum TNF-α concentrations decreased to near-undetectable levels within 2 h of commencing AZD9773 infusion and maintained in most patients for the duration of treatment	Safety, PK, and PD data support evaluation of AZD9773 in larger phase IIb/III studies	^409^
		2014	300	Phase IIb RCT	AZD9773	Mean number of ventilation-free days did not differ between groups. Comparable results were observed for other endpoints, such as mortality rates and relative risk of death.	Though AZD9773 rapidly and efficiently decreased plasma TNF-α concentration in severe sepsis/septic shock, beneficial effect could not translate into clinical significance	^410^
Platelet-activating factor (PAF) antagonist	Platelet-activating factors and structurally related oxidized phospholipid are important pro-inflammatory mediators of systemic inflammation during sepsis. PAF antagonist specifically degraded PAF and oxidized phospholipid to inactive metabolites	2000	152	Phase II RCT	Lexipafant (BB-882)	No benefits on survival, hemodynamic status, respiratory function, or organ failure scores		^411^
		2000	98	Phase II RCT	TCV-309	No effect on overall mortality. However, a substantial reduction in organ dysfunction and morbidity, frequently associated with septic shock was achieved		^412^
		2003	127	Phase IIb RCT	rPAF-AH	rPAF-AH was well tolerated in sepsis patients. An improved 28-day mortality rate and reduced incidence of multiple organ dysfunction were observed in the low rPAF-AH dose group	No treatment-related deaths were observed. Adverse events are comparable between rPAF-AH- and placebo-treated patients	^413^
		2004	1425	Phase III RCT	rPAF-AH	No effect on mortality reduction		^414^
Toll-like receptor-4 antagonists/inhibitors	Toll-like receptor-4 antagonists/inhibitor interferes with signal transduction mediated through the CD14–TLR-4 complex and suppresses elevation in serum TNF-α, IL-1, and IL-6	2010	274	RCT	TAK-242	Lack of effect of TAK-242 in suppressing serum IL-6 level had led to termination of trial. Though mortality risk is reduced in subset of patients with shock and respiratory failure with high-dose TAK-242, the differences were not significant	TAK-242 failed to suppress cytokine levels. Non-significant mortality reduction is observed in subset of patients with shock and respiratory failure	^415^
		2010	300	Phase II RCT	Eritoran	Eritoran was well tolerated among sepsis patients. Non-significant mortality reduction is observed. In the eritoran-treated group, a trend toward lower mortality rate is observed in patients with severe sepsis and high predicted risk of mortality, while a trend toward higher mortality rate for patients with lowest APACHE II scores	This trend in mortality rate support the need for future trials that are powered to demonstrate beneficial outcome in patients with high mortality risk	^416^
		2013	1961	Phase III RCT	Eritoran	No effect on mortality reduction		^409^
Programmed cell death ligand 1 monoclonal antibody (PD-L1)	Increased expression of PD-1 and PD-L1 were often seen in patients with sepsis, which is associated with T cell exhaustion. Blockage of PD-L1 by nivolumab is potent to reverse T cell apoptosis and recover immune cell receptor function, therefore preventing immunosuppression	2020	13	Phase I/II RCT	Nivolumab	Nivolumab seemed to improve sepsis-induced immunosuppression over time		^417^

**Table 2:** Comparison of therapeutic effect on biological agents targeting on coagulation cascades

Anticoagulant therapy	Trial	Year	Study design	Population	Subjects	Results	Source
Recombinant activated protein C (drotrecogin alfa)	PROWESS	2001	Prospective RCT	1690	Severe sepsis	Significant mortality reduction (6.1%, *p* = 0.05) with increased risk of bleeding complication	^321^
	PROWESS post hoc	2004	PROWESS post hoc	1690	Severe DIC sepsis patients (including overt DIC)	Trend toward greater mortality reduction in DrotAA-treated overt DIC patients compared with those without	^418^
	ENHANCE	2005	Single arm trial compared with PROWESS	2434	Severe sepsis	Comparable mortality reduction to PROWESS. Significant lower mortality rate (*p* = 0.01) for patients treated within 24 h	^419^
	ADDRESS	2005	Prospective RCT	2640	Severe sepsis with low risk of death (APACHE <25 or single organ failure)	No effect. Higher incidence of serious bleeding complication	^420^
	XPRESS	2007	Prospective RCT	1994	Severe sepsis patients treated with drotreocogin alfa	Coadministration of prophylactic heparin does not cause increase in mortality rate and is safe in patients receiving DroAA	^421^
	RESOLVE	2007	Prospective RCT	477	Severe sepsis children	No effect	^422^
	N/A	2013	Prospective RCT	411	Septic shock	No effect	^423^
	PROWESS-SHOCK	2012	Prospective RCT	1697	Septic shock	No effect	^424^
	N/A	2012	Meta-analysis	6781	Severe sepsis and septic shock	No effect. Clinical use is prohibited for severe sepsis patients outside RCT	^425^
Recombinant human tissue factor pathway inhibitor (tifacogin)	OPTIMIST	2003	Prospective RCT	1754	Severe sepsis with high INR	No effect	^426^
	CAPTIVATE	2010	Prospective RCT	2138	Severe CAP	No effect	^427^
Recombinant human thrombomodulin (ART-123)	Saito	2007	Prospective RCT	234	DIC in hematologic malignancy and infectious patients	Significant higher DIC resolution rate with reduced bleeding symptoms. No significant effect on mortality reduction	^428^
	Aikawa	2010	Retrospective subgroup analysis of Saito study	80	DIC in infectious patients	Significant mortality reduction for patients recovered from DIC than in those who did not recover from DIC, and this effect is more pronounced in the TM-a group	^429^
	Vincent	2013	Prospective RCT	741	Sepsis and suspected DIC patients	Efficacy and safety was proven. Post hoc analysis showed greatest survival benefits in sepsis patients presented with organ failure and coagulopathy (prolonged INR and reduction of platelet count)	^430^
	Hagiwara	2016	Prospective RCT	92	Severe septic-induced DIC	Improved DIC recovery and lower relative changes in D-dimer baseline levels (*p* = 0.001). No significant effect on mortality reduction	^431^
	SCARLET	2019	Prospective RCT	800	Patients with sepsis-associated coagulopathy	No significant effect on mortality rate for patients with sepsis-associated coagulopathy	^432^
	N/A	2019	Meta-analysis	1762	Sepsis-induced coagulopathy	Moderate evidence for mortality reduction	^433^
	SCARLET post hoc	2020	Post hoc subgroup analysis of SCARLET	800	Patients with sepsis- associated coagulopathy	Modest effect. For rhTM-treated patients having higher baseline thrombin generation biomarker levels, higher mortality reduction is observed compared to the placebo group	^434^
Recombinant antithrombin (rAT)	KyberSept	2001	Prospective RCT	2314	Severe sepsis	No effect. Survival benefits on subgroup of patients without concomitant heparin	^435^
	KyberSept post hoc	2006	Retrospective post hoc analysis of the KyberSept study	563	Severe sepsis DIC patients treated with high-dose AT without concomitant heparin	Significant mortality reduction in DIC patients treated with high-dose AT without concomitant heparin (*p* = 0.02), but not in patients without DIC	^436^
	JAAMDICAT	2013	Prospective RCT	60	Sepsis DIC patients with low antithrombin levels	Improved DIC recovery. No effect on mortality reduction	^146^
	Iba Toshiaki	2017	Retrospective study	159	Sepsis DIC with low AT levels	Coadminstration of rTM improve survival rate in AT-treated sepsis DIC patients without increased risk of bleeding	^437^
	N/A	2016	Meta-analysis	2858	Severe sepsis DIC patients	No effect	^438^
	N/A	2018	Revisited meta-analysis	766	Severe sepsis DIC patients	Replacing the mixed sepsis patient group with only those confirmed with DIC according to a post hoc analysis, reanalysis showed significant reduction in mortality risk	^439^
Heparin	HETRASE	2009	Prospective RCT	319	Sepsis patients	No effect	^440^
Efficacy of anticoagulant therapies	N/A	2016	Network meta-analysis for three specific population with sepsis	14,767	Sepsis patients, sepsis-induced coagulopathy, and sepsis-induced DIC patients were separately analyzed	No survival benefits for overall sepsis and sepsis-induced coagulopathy. Significant mortality reduction in population with sepsis-induced DIC	^441^
	N/A	2016	Post hoc analysis of retrospective registry	2663	Sepsis patients	Significant mortality reduction in patients with sepsis-induced coagulopathy and very severe disease (high SOFA score)	^442^
	N/A	2018	Network meta-analysis	1340	Severe sepsis DIC patients	No significant reduction in mortality and bleeding risks for all selected anti-coagulants. Antithrombin is associated with higher DIC resolution rate	^443^

Lately, post hoc studies have observed encouraging results after stratification and subgroup analysis (Tables 1 and 2). In common, these recent studies have taken advantages on revised inclusion criteria based on novel biomarkers or scoring system consisted of numerous clinical variables, therefore emphasizing the importance of precise therapy and effectiveness of biomarker-guided trials. Before that, several studies have already proposed potential biomarkers for endothelial injuries, including fragments of membrane-bound molecules generated after proteolytic cleavage by neutrophils. Administration of recombinant (i.e., TM) or activated form (i.e., APC) of molecules showed uplifting results in early clinical trials (Table 2); however, have also risen certain concerns on lethal adverse events, such as bleeding complications. Monitoring of vast group of biomarkers associated with innate immune responses, receptor expression, vascular barrier integrity, and tissue/organ injuries could provide indirect evidences informing the real-time magnitude and depth of dysregulated host responses. Dynamically reflecting the immune homeostasis status relevant to clinical course are essential for elucidating sepsis pathophysiology. Innate immune response biomarker such as DAMPs, PAMPs, chemokines, and cytokines are capable for guidance of therapeutic strategies targeting on removal and inhibition of respective molecular stimuli. These approaches might be beneficial for early reversal of detrimental events. With the help of other novel biomarkers, continuous monitoring levels of immune cell activity help to identify patients who might benefit most from a specific intervention. Such approach is vital especially when majority of sepsis therapeutic strategies might be harmful to patients who express immunoparalysis or a more dysregulated proinflammatory host response. Monitoring of biomarkers related to vascular, tissue, and organ failure could early identify patients suspected of severe sepsis and refractory septic shock. These measures have provided rationales for future biomarker-guided therapy and personalized, tailored therapy.

Concurrently, significant number of studies have identified previously unknown components and novel relationship between signaling pathways that serve as key drivers for enhanced or dysregulated inflammatory responses. These studies inspired therapeutic approaches that target on a wider scope of host responses and shed light on the development of novel intervention therapies. Asides from the well-known PAMPs such as LPS and mannose, identification of a substantial group of DAMPs showed superior role in sepsis pathogenesis. Inhibition of ligands or receptors could prevent activation of devastating downstream events. These referred to the use of monoclonal antibodies, downregulation of DAMP expression, and inactivation of the intrinsic binding interaction (Table 3 and Fig. 5).

**Table 3:** Novel therapeutic molecules, targeted mechanism, and their clinical impact in sepsis

Therapeutic molecules	Targeted mechanism	Therapeutic strategies	Model used	Special features	Clinical impact	Source
Kukoamine B (KB)	LPS- and CpG-DNA-induced pro-inflammatory responses	Traditional Chinese herbs	Heat-killed *E. coli* challenged sepsis models	As a dual inhibitor of LPS and CpG-DNA, KB directly neutralize and prevent them from interacting with macrophages	Inhibit expression of pro-inflammatory mediators without interfering with signal pathways or cell viability of macrophages. Protect mice from *E. coli* challenged and reduced circulatory LPS and TNF-α	^444^
Ceria–zirconia nanoparticles (7 CZ NPS)	Regeneration of anti-oxidants	Nanotherapeutic approach	CLP-/LPS-induced sepsis models	Ceria nanoparticles are potential regenerative antioxidants that could overcome the constantly produced ROS in a single dose. Zr^4+^ facilitate regeneration of Ce^3+^ and higher Ce^3+^/Ce^4+^ ratio so as to exhibit best ROS scavenging performance during sepsis	7CZ NPS block aberrant inflammatory responses via ROS scavenging and have additional effects on inflammatory pathway. Expected to be internalized by macrophages and neutrophils, these strongly phagocytic immune cells infiltrate to damaged tissues and act directly with intracellular ROS at infection focus. Overall, it demonstrated reduced mortality and systemic inflammation in sepsis models	^395^
Nucleic acid–scavenging nanoparticles (NABNs)	Inhibit cfDNA-TLR9-MyD88-NF-κB pathway	Nanotherapeutic approach	CLP-induced sepsis models	Biodegradable mesoporous silica nanoparticles with different charge density serve as cfDNA scavengers and are also known as nucleic acid–scavenging nanoparticles (NABNs). High cfDNA is strongly associated with sepsis mortality	With favorable accumulation and retention behavior in inflammatory tissues, NABNs inhibit cfDNA-induced inflammation and improve multiple-organ injury and mortality caused by sepsis	^396^
Xuebijing	HMGB1-mediated inflammatory pathway	Traditional Chinese medicine	CLP-induced lung injury model	Xuebijing downregulate HMGB1 and RAGE expression and interaction, thereby reduce cytokine-mediated inflammation (TNF-α, IL-1β, IL-6) and neutrophil infiltration in the alveolar space	Inhibit HMGB-mediated inflammation and improve survival	^445^
TN domain-specific mAb	HMGB1-mediated inflammatory pathway	Monoclonal antibody	CLP-induced sepsis models	TN, an endogenous protein, is capable to capture HMGB1 to trigger several conflicting events during sepsis: endocytotic degradation of HMGB1, macrophage pyroptosis, attenuated release of HMGB1, and induction of beneficial chemokine secretion. Sepsis-induced TN depletion showed pathogenic consequences while supplement of subphysiological TN confers protection	TN-specific protective monoclonal antibody bind and inhibit TN/HMGB1 interaction, prevent endocytosis of HMGB1, and attenuate sepsis-induced TN depletion and tissue injury, rescuing animals from lethal sepsis	^393^
CD28 T-lymphocyte receptor mimetic (Reltecimod, AB103)	Interaction between superantigen and costimulatory receptor CD28 dimer expressed on T cells, which function to mediate T helper type 1 cytokine responses	Monoclonal antibody	*E. coli* LPS-induced, CLP-induced sepsis models	Receipt of AB103 attenuated inflammatory cytokine responses (TNF-α, IL-6) and neutrophil influx into tissues and promoted bacterial clearance	AB103 resulted in higher survival rate and reduces mortality in experimental models of polymicrobial and Gram-negative bacterial infection and sepsis	^446^
			Surgical confirmation of necrotizing soft tissue infections (NSTI) and organ dysfunction (mSOFA)	Early administration of reltecimod resulted in improvements of 28-day survival, day 14 mSOFA, and avoidance of surgical procedures	Improve resolution of organ dysfunction and hospital discharge status	^447^
ALK inhibitor (LDK378)	A novel ALK-EGFR-AKT pathway responsible for regulation of STING-dependent inflammatory responses	FDA-approved anticancer drug	CLP-/LPS-induced sepsis models	ALK pathway was upregulated in sepsis patients. ALK-EGFR-AKT pathway is a critical driver of STING activation. Pharmacologically blocking the ALK-dependent STING signaling pathway could modulate the DNA-induced excessive inflammation response in sepsis, particularly referring to bacterial CDN or host DNA released by injured cells	LD378 confers protection against lethal sepsis by alleviating tissue injuries and pro-inflammatory cytokines. LD378 also improve micro-circulation and macro-circulation and reduce organ injuries	^359^
Sialic acid–binding immunoglobulin-like lectin-E nanoparticles decorated with natural Siglec ligand	Siglec-E induction pathway provide inhibitory signal for TLR-mediated response in macrophages and neutrophils	Nanotherapeutic approach	CLP-induced sepsis models	Siglec system have important roles in homeostasis and restoration of normal immune cell function. In inflammatory settings, LPS enhance IL-10 production, which induce Siglec-E expression that forms a positive feedback loop for IL-10 expression	Sialylated nanoparticle promote oligomerization and activation of Siglecs on macrophages, reduce inflammatory cytokine production in models of systemic inflammation and localized lung injury	^448^
Magnetic nanobeads coated with an engineered human opsonin	Extracorporeal removal of pathogen and endotoxin from bloodstream	Nanotherapeutic approach	*Staphylococcus aureus* or *E. coli*-infected rats	Blood flow from an infectious patient was mixed with nanobeads incorporated in this extracorporeal blood cleansing device coated with an engineered human opsonin, the magnetic nanobeads capture and remove opsonin-bound pathogen and toxin from the blood, while cleansed blood is then returned back to individuals	In models of endotoxemic shock, this biospleen device cleared >90% bacterial from blood, reduced pathogen and immune cell infiltration in multiple organs, and decreased inflammatory cytokine levels	^394^
pH/enzyme-responsive bioresponsive nanocarrier targeted for drug delivery to infectious microenvironments (IME)	Vascular cell adhesion interfere with leukocyte recruitment responses and various inflammatory signaling pathway at the infectious microenvironments (IME)	Nanotherapeutic approach	*P. aeruginosa* challenged acute peritonitis model	Biotin–avidin system facilitate conjugation of ICAM antibody to nanoparticles. Together with pH and enzyme-responsive properties, these facilitate co-delivery of antibiotics and anti-inflammatory agent to IME, thus effectively control both bacterial burden and host inflammatory response	Co-delivery of antibiotics and anti-inflammatory agent to IME improved survival rate in animal models, observed with marked reduction in leukocyte counts, inflammatory cytokines (TNF-α, IL-1β, and IL-6), and CFU detected in peritoneal fluids and blood. Proteins content were also reduced in peritoneal fluids, suggesting leaky vasculature repairing	^397^
An ANG2-binding antibody (ABTAA)	Angiopoietin-TIE2 system	Monoclonal antibody	CLP-/LPS-induced sepsis model	Ang2, a Tie2 antagonist, is often elevated in severe sepsis patients. By inducing Ang2 oligomerization and clustering at Tie2 receptor molecule, ABTAA covert the deleterious antagonist into a novel Tie-2 activator and induce downstream endothelial protective signaling. Converting existing Ang2 into agonist of Tie2 is a more effective approach than blocking with an Ang2 inhibitory antibody or supplement of Tie2 agonist Ang1	ABTAA augment survival by strengthening endothelial glycocalyx, reducing cytokine storms, preventing vascular leakage, and mitigating organ damage via barrier defense effect of TIE2 activation	^377^
Adrenomedullin-binding antibody (Adrecizumab, ADZ)	Inhibition of sepsis-induced ADM to stimulate protective effect by restoring endothelial function	Monoclonal antibody, biomarker-guided therapy	Early septic shock with high concentration of circulating ADM (recruiting)	ADM is a vasoactive peptide expressed within endothelial cells. Described as a double-edged sword, ADM could not only induce vasodilation and hypotension but also reinforce endothelial barrier. During sepsis, high plasma concentrations of ADM were correlated with worse clinical outcomes	Experimentally, AZM shift distribution of ADM away from interstitium toward blood and facilitate interaction with endothelial cell receptors to prevent vascular leakage and hypotension. In preclinical studies, ADZ improved vascular barrier function, reduced vasopressor demand and organ dysfunction, and improved survival. Phase II study was completed and pre-results will be published shortly	^449^
Human recombinant alkaline phosphatase	Dephosphorylation of endotoxin and adenosine triphosphate by alkaline phosphatase resulted in an attenuated inflammatory response	Monoclonal antibody	Sepsis patients with acute kidney injury (AKI)	As an endogenous enzyme, alkaline phosphatase exhibits detoxifying effects through dephosphorylation of endotoxins and pro-inflammatory mediators, such as extracellular adenosine triphosphate	In critically ill sepsis-associated AKI patients, alkaline phosphatase improves endogenous creatinine clearance but did not have significant effect on short-term kidney function (first week)	^450^

**Fig. 4:**
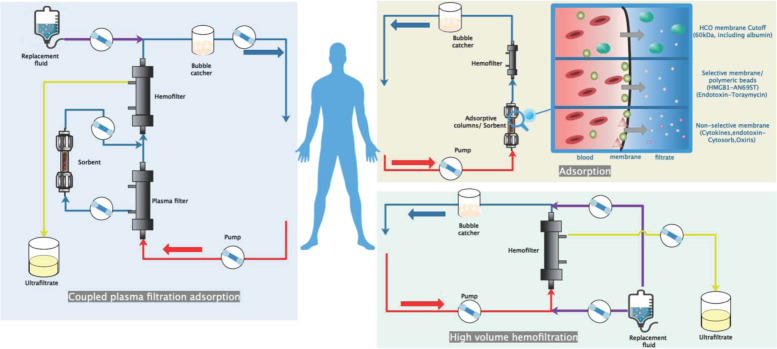
Schematic diagram of different types of extracorporeal blood-purification strategies (removal of mediators). Red line = arterial line, blue line = venous line, yellow line = ultrafiltrate, purple line = replacement fluid

Systemic removal of pro-inflammatory mediators is another effective therapeutic approach that has been extensively applied in clinical scenarios. Extracorporeal removal of inflammatory mediators or toxic substances consists of conventional and novel blood purification techniques. Essential information of each of these techniques were compared and presented in table form (Table 4 and Fig. 4). In terms of flow volume and modality, these could be classified into high volume hemofiltration and continuous hemofiltration,^386^ while more studies shed light on the type of CRRT membrane or adsorption cartridges incorporated,^387^ or the use of coupled plasma filtration adsorption or double adsorption.^388^ Clinically, polymyxin B-immobilized hemoperfusion serves as an adjuvant therapy for septic shock by removing circulating endotoxin via absorption.^389^ CytoSorb, a CE-approved cytokine adsorber, was recently under great concerns due to its safe rescuing effect on SARS-CoV-2 infected multiple organ dysfunction syndrome patients.^390^ As an extracorporeal cytokine hemoadsorption device, CytoSorb showed significant improvements on norepinephrine requirements and PCT but failed to obtain benefits on clinical outcomes.^391^

**Table 4:** Extracorporeal techniques for blood purification in sepsis

Type of techniques	Mechanism	Published year	Study type	Population	Study subjects	Clinical outcomes		Comments	Source
						Primary	Secondary		
Short term high-volume hemofiltration (HVHF)	Capable of maintaining azotaemic, acid–base, and fluid homeostasis and removal of toxins and inflammatory mediators, experimental studies showed that HVHF can improve myocardial performance and systemic hemodynamics while also removal of cytokines. Clinical studies conducted in refractory septic shock also observed similar results	2006	Prospective nonrandomized study	20	Severe septic shock patients unresponsive to multi-intervention approach in surgical unit	NE requirements and perfusion parameters	Feasibility, tolerance, and adverse events	As a salvage therapy, HVHF might be beneficial in septic shock patients	^451^
		2008	ACT, Phase III RCT	1124	Critically ill AKI patients with failure of at least one nonrenal organ failure or sepsis patients in need of renal replacement therapy	60-day mortality rate	In-hospital death and recovery of kidney function	No significant improvements	^452^
		2013	IVOIRE, Phase III RCT	137	Critically ill patients with septic shock and AKI at early phase	28-day mortality rate	SOFA, hemodynamic profile, mechanical ventilation, prognostic outcome	No significant improvements	^453^
		2014	Meta-analysis	241	Sepsis or septic shock patients	28-day mortality rate	Recovery of kidney function, lengths of stay, vasopressor dose reduction. and adverse events	Insufficient evidences support therapeutic benefits for routine use of HVHF for septic AKI	^454^
		2017	Meta-analysis	200	Severe sepsis and septic shock patients	Mortality rate	Length of stay, severity of organ dysfunction, adverse events	Studies on the use of HVHF in critically ill patients with sepsis or septic shock remains scarce	^455^
Continuous venovenous hemofiltration (CVHF)	With benefits from early application of short-term HVHF observed in septic shock patients, the classic CVVH technique was also suggested to limit inflammation in septic shock	2009	Phase III RCT	80	Severe sepsis patients with organ failure at early phase	Number, severity, and duration of organ failures	14-day mortality rate and time to death	Early application of CVVH is deleterious in severe sepsis patients	^456^
Hemodialysis with high cut-off membrane (HCO)	Designed with larger pore size, HCO membrane application with standard RRT modality showed greater cytokine removal capacity but with albumin loss	2006	Pilot study	30	Sepsis-induced acute renal failure	N/A	N/A	HCO hemofiltration exert beneficial effects in terms of reduction in vasopressors dose and clearance for IL-6 and IL-1ra	^457^
		2007	Phase I RCT	10	Septic patients with acute renal failure	Relative change in plasma IL-6 concentrations	Relative change in other plasma cytokines and albumin	High cut-off intermittent hemodialysis achieved both uremic control and diffusive cytokine clearances and a greater relative decrease in plasma cytokine concentrations	^458^
		2016	Retrospective study	16	Sepsis patients with AKI	N/A	N/A	Significant improvements on ICU mortality, days of vasopressors, and mechanical ventilation	^459^
Polymyxin B hemoperfusion (PMX-HP/Toraymycin)	As a cyclic cationic polypeptide antibiotic, polymyxin B (PMX) showed high affinity for endotoxin. When immobilized into polystyrene fibers in a hemoperfusion device, PMX function to remove circulating endotoxin by adsorption and modulate dysregulated host responses without nephron toxicity	2009	EUPHAS, Phase III RCT	64	Severe sepsis or septic shock from intra-abdominal Gram-negative infections	Change in MAP and vasopressor requirement	28-day mortality rate, SOFA score, PaO_2_/FIO_2_	Significant improvement in hemodynamics, organ dysfunction, and reduced 28-day mortality	^460^
		2015	Abdomi, Phase III RCT	243	Postoperative intra-abdominal septic shock patients	28-day mortality rate	SOFA score, time to withdraw catecholamine, adverse events	No significant improvements	^461^
		2018	EUPHRATE, Phase III RCT	450	Septic shock patients with high endotoxin activity (EAA ≥ 0.60)	28-day mortality rate	N/A	No significant improvements	^462^
		2017	Propensity-matched cohort study	522	Septic shock patients	All-cause hospital mortality	ICU mortality and number of ICU-free days (ICUFDs) in the first 28 days	PMX-HP reduce all-cause hospital mortality and length of ICU stay	^389^
		2018	Post hoc analysis of EUPHRATES	450	Septic shock patients with high endotoxin activity (EAA ≥ 0.60)	28-day mortality rate	Changes in MAP, cumulative vasopressor index, EAA reduction, and other prognostic outcomes	Patients with septic shock and high EAA experience reduced 28-day mortality rate and improvements in other prognostic outcomes	^463^
		2018	Meta-analysis	857	Critically ill adult patients with sepsis or septic shock	N/A	N/A	No significant benefit for 28-day mortality and organ dysfunction scores	^464^
Polymyxin B hemoperfusion (PMX-HP) with recombinant thrombomodulin (rTM)	PMX-HP directly adsorb endotoxin and activated mononuclear cells (cytokines) so as to improve hemodynamics. rTM exerts not only anticoagulation but also anti-inflammatory properties via anti-HMGB1 activity. Combination of PMX-HP and rTM is expected to block the vicious cycle of a cytokine storm	2013	Historical controlled trial	22	Septic shock associated with DIC	60-day survival rate	SOFA score	60-day survival rate is significantly higher in the treatment group	^465^
		2020	Post hoc analysis from the dataset of Japan septic DIC registry	2350	Sepsis patients who were given combination therapy of PMX-HP with ART123	Hospital mortality	N/A	Significant beneficial effect modification on survival outcome was observed from patients coadministrated with ART-123 and PMX-HP	^466^
Continuous hemodiafiltration using a PMMA membrane (PMMA-CHDF)	PMMA-CHDF remove cytokines from blood continuously and efficiently by adsorption to membrane matrix of a hemofilter	2008	Observational cohort study	43	Septic shock patients	N/A	N/A	Significant improvements in hemodynamics, urine output, hypercytokinemia, and dysoxia	^467^
		2011	Comparative study	30	Septic shock patients with acute renal failure	28-day survival	Hemodynamic profile	Compared with polyacrylonitrile membrane CHDF, PMMA-CHDF-treated patients have improved hemodynamics and 28-day survival	^468^
AN69ST	AN69ST membrane has excellent cytokines adsorption capacity. Surface treatment (ST) with polyethyleneimine and heparin allow greater bulk adsorption (HMGB1)	2014	Randomized, prospective, single-center trial	34	Septic shock patients	28-day mortality rate	Sequential changes in lactate, cytokine blood levels, and hemodynamics	Significant reduction in IL-6 and lactate levels were observed, though APACHE II score and 28-day mortality were high	^469^
		2017	Retrospective observational study	156	Critically ill patients receiving CRRT with AN69ST membrane	In-hospital mortality	ICU length of stay	CRRT with AN69ST membrane is associated with better in-hospital mortality	^470^
Modified AN69ST membranes (Oxiris)	Compared with AN69ST, modified coated layer allowed additional adsorption of endotoxin and cytokines with better anticoagulation properties	2019	Randomized trial	16	Septic shock-associated acute renal failure requiring CRRT	Change in plasma endotoxin	Change in cytokine levels	Effective removal of endotoxin and TNF-α, IL-6, IL-8, and IFN-γ in patients with septic shock-associated acute renal failure	^471^
		2019	Observational study	31	Septic shock patients requiring CRRT	Hospital mortality	SOFA score, norepinephrine reduction, hemodynamic, respiratory, and biological parameters	Higher survival was observed than predicted. Hemodynamic status and lactatemia were improved in intra-abdominal sepsis and Gram-negative infections	^472^
Coupled plasma filtration adsorption (CPFA)	CPFA is a complex circuit that includes a hemofilter, a plasma filter, and an adsorbing cartridge, which requires an adequate balance of flows, dilutions, and anticoagulation. It is an extracorporeal technique intended to non-selectively remove the majority of soluble inflammatory mediators from systemic circulation	2014	COMPACT-1, phase III RCT	192	Septic shock patients	Mortality at discharge from hospital	90-day mortality, new organ failures and ICU-free days within 30 days	No significant improvements in mortality or other prognostic outcomes. Subgroup suggested survival benefits when high volume plasma is treated. CPFA is not encouraged in everyday clinical practice	^388^
		2015	Meta-analysis	242	Severe sepsis patients	28-day mortality	Mediator adsorption, mean arterial BP and oxygenation ratio	Evidence for CPFA in severe sepsis is sparse and of poor quality. However, improvements in survival rate is noted in this meta-analysis	^473^
		2020	Retrospective study	39	Severe burn septic shock in need of renal replacement therapy	N/A	N/A	CPFA treatment for burn patients with AKI-RRT and septic shock was associated with lower mortality rate compared to CRRT alone	^474^
Cytosorb	Containing hemocompatible porous polymeric beads, Cytosorb is capable for removal of cytokines (IL-1 β, IL-6, IL-8, IL-10, and TNF-α) and mild molecular weight toxins (myoglobin, hemoglobin, bilirubin, bile acid, toxin etc.)	2017	Prospective single-center study	20	Refractory septic shock	Noradrenaline requirements	SOFA scores, resolution of shock and lactate clearance	Significant improvements in noradrenaline reduction, lactate clearance, SOFA scores, shock reversal achievement, and 28-day survival	^475^
		2017	Phase II RCT	97	Sepsis or septic shock with ARDS	Normalized IL-6 serum concentration before and after treatment	Ventilation time, normalized levels of other cytokines, vascular endothelial growth factor (VEGF), CD4+ T cell activation, 28-day mortality, P/F ratio, and multiple organ dysfunction score	Hemoadsorption removed IL-6 but did not lead to lower plasma IL-6 levels. No significant improvement for organ dysfunction score, ventilation time, and time course of oxygenation	^476^
		2019	Pilot trial	20	Early-onset sepsis or septic shock with ARDS	N/A	N/A	Safe with significant improvements on norepinephrine requirements, PCT, and Big-endothelin-1 concentrations	^391^
		2021	Prospective observational multi-center study	45	Sepsis and septic shock	Change in MAP, vasopressor reduction	SOFA and APACHE scores, vital parameters	CytoSorb might be an effective adjunctive therapy in sepsis and is advised to start at an early stage	^477^

**Fig. 5:**
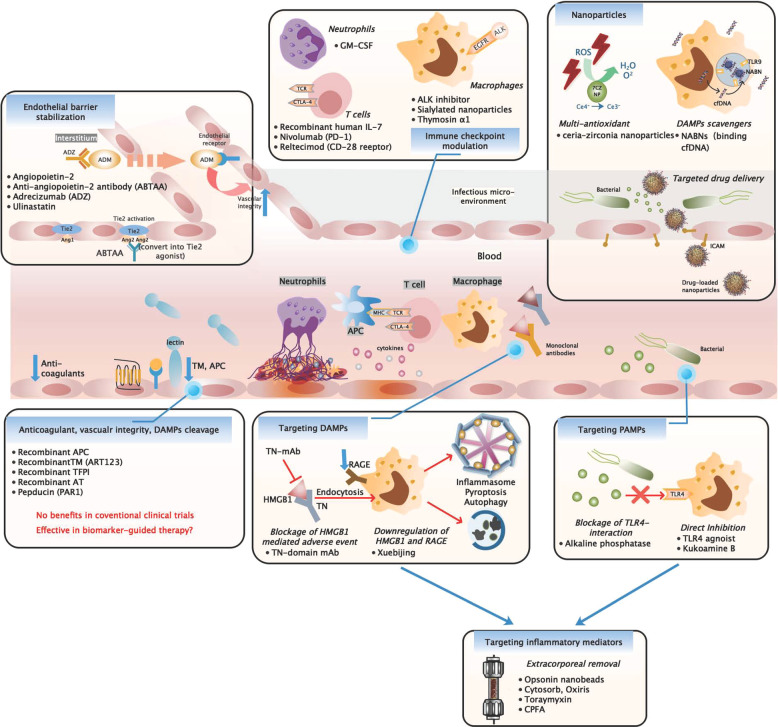
Novel intervention therapeutic strategies in sepsis. In terms of the signaling pathways or molecules targeted, potential therapeutic strategies for sepsis that have been proposed so far could be classified into six categories: (1) targeting DAMPs (including host cell stress), (2) targeting PAMPs, (3) targeting inflammatory mediators (anti-inflammatory), (4) immune checkpoint modulation, (5) endothelial barrier stabilization, and (6) restoration of vascular anticoagulant properties. Nanoparticles therapy is a promising novel strategy that have broad therapeutic effects in sepsis experimental studies. Titles in italic refer to the specific targeted mechanism (strategies). Dot-labeled subtitles refer to the specific therapeutic techniques or bioactive molecules proposed

Recently, the novel ALK-EGFR-AKT pathway that serve as potential regulator on STING-dependent inflammatory responses was suggested to be useful therapeutic target during sepsis. LDK378, a USA-FDA approved ALK inhibitor, was shown to exhibit anti-inflammatory effect and potent to confer protection against lethal endotoxemia and sepsis in mice.^359^ Other approach that interfere with interaction between toxins/alarmins and their reciprocal receptor also result in attenuated cytokine gene expression and inflammatory responses. CD28 homodimer is proven to prevent access of superantigen to CD28 expressed on T cells,^392^ while TN-domain-specific mAb blocking the harmful interaction between TN and HMGB1 has prevented macrophage pyroptosis and rescued animals from lethal sepsis^393^ (Table 3 and Fig. 5).

Biomedical nanoparticles posing specific binding affinity and catalytic property are powerful therapeutic agents that have broad therapeutic effect on sepsis. Decorated or coated with specific ligands, these nanoparticles were designed to have preferential effect on various targeted substrates. Nanobeads coated with human opsonin mannose-binding protein captured pathogen and toxin, which would in turn minimize immune cell infiltration in multiple organs,^394^ while ceria–zirconia nanoparticles and silica nanoparticles that serve as specific scavengers for ROS and nucleic acid, respectively, were targeted to reduce stress-induced injuries and cfDNA-induced inflammation.^395,396^ Nanoparticles also serve as an ideal carrier for targeted drug delivery to infectious microenvironments (IME). Co-delivery of antibiotics and anti-inflammatory agent to IME markedly reduce inflammatory responses and improve survival rate^397^ (Table 3 and Fig. 5).

### Future prospectives and conclusion

Immunological pathologies and pathogenesis of sepsis is becoming gradually clear; however, the complete outlook of these mechanisms still could not reach agreement due to the difficulties to identify and clarify clinical significance of observed host responses. This situation could be even more complex as heterogeneous outcomes were observed between individuals or among different host organs. Sepsis remains a huge challenge to intensive care specialist with a lot of unsolved issues. Mutual goal of sepsis management is to early identify and provide rapid intervention, so as to prevent development of irreversible septic shock and multiple organ dysfunction syndrome. However, complexity of this syndrome makes it impossible to develop a precise golden standard for routine use. With breakthroughs in identifying sepsis-specific biomarkers and involved signaling pathways, more precise and accurate diagnostic measures are required to rule out predisposed sepsis patients and to allocate precise therapeutics in advance, before shifting the equilibrium to irreversible detrimental outcomes. Future studies should focus on molecular or biomarker-based therapy. Also, recent studies have proposed the observed heterogeneous host responses to be related to genomic profile between individuals, which is referred to as sepsis endotypes.^398^ Pronounced alteration of RNA transcripts related to inflammation and mitochondria dysfunction was distinctly observed in sepsis patients and independent of the source and causative pathogens.^399,400^ Such altered genomic profile was also found in burn injuries, traumatic, and critically ill patients.^401^ Genetic variation might have determined the leukocyte transcriptome and degree of immunosuppression in sepsis patients.

This review focused on dysregulated/altered signaling pathways and molecules involved with sepsis pathogenesis. Any statements of perspectives were based on information from hitherto described sepsis-related signaling pathways in previous studies. Potential molecular mechanism for sepsis pathogenesis as well as cross-talks between these pathways were also discussed in detail. Proposed outlook of these activated host responses and therapeutic potentials were illustrated for better understanding. Lastly, informative tables were used to compare clinical efficacy of RCTs and valuable studies related to conventional or novel therapeutic strategies targeting on signaling pathways and molecules.
